# Supplementary material for: Evaluation of short-term multi-target respiratory forecasts over winter 2024-25 in England using sub-ensemble contribution analyses
Source: PLoS Comput Biol. 2026 Aug 12;22(8):e1014644. doi: 10.1371/journal.pcbi.1014644 (PMC13506087; doi:10.1371/journal.pcbi.1014644)
Supplement: Supplementary file 1 [file pcbi.1014644.s034.pdf]

Review of "Evaluation of short-term multi-target respiratory forecasts over winter 2024- 25 in England using sub-ensemble contribution analyses"

March 2026

1 Introduction

1.1 Manuscript summary

This manuscript assesses the performance of ensemble forecasts for COVID-19 and influenza hospitalizations in England. In particular, the manuscript analyzes the contributions of individual constituent forecasting models to overall ensemble forecast performance, with the aim of providing information that could improve future forecast ensembles.

Each week during the 2024-2025 respiratory virus season (autumn through spring, with a break during the holiday season, modelers from the United Kingdom Health Security Agency (UKHSA) provided forecasts of daily new incident hospitalizations with COVID-19 and influenza over the coming two weeks. They also provided a categorical "trend" forecast for the coming two weeks based on these forecasts of daily counts. They provided these real-time forecasts to stakeholders including the UK National Health Service (NHS).

The UKHSA forecasts were "'ensembles" of multiple individual forecasting models. Probabilistic predictions from each individual model are aggregated in a Bayesian framework ("posterior stacking") to produce overall posterior predictive distribution to which each model contributes equally.

As I understand it, the scientific aims of the manuscript are:

1. Assessing ensemble forecast performance and reliability for the "operational ensemble" produced and shared in real time.
2. Assessing the impact of individual constituent models on that ensemble's forecast performance, including how consistently and to what degree models consistently improved or degraded ensemble forecast performance.

3. Determining to what degree there are tradeoffs in ensemble design for count forecasts versus trend forecasts.

1.2 Summary of evaluation

The scientific work conducted here is important. Retrospective forecast evaluation is critical, especially when forecasts will be used to inform high-impact decisions. The fact that these infectious disease forecasts were produced by a public health agency and shared with a national healthcare system makes this particular forecast evaluation of great interest. Another strength of the work is that this retrospective evaluation is aimed at improving ensembles (by investigating methods for determining a good set of individual component models). I believe that this manuscript should ultimately be published.

That said, I think the manuscript needs to be revised and expanded with additional detail in order to be persuasive. Both the methods and the results are missing information that I believe should be included. My principal critiques are:

1. Insufficient detail on individual models limits the reader's ability to interpret key findings (2.1)
2. Only a small subset of the forecasts and scores analyzed are visualized or otherwise reported in the manuscript (2.2)
3. Key methodological choices made in the analysis are unclear or should be justified more (2.3)

I explain these critiques below. I also provide some minor comments and suggestions.

1.3 Alternative possible framing

I have assessed the manuscript principally as it does or does not achieve the three scientific aims I enumerated above. That said, there is another potential interpretation of the manuscript's scientific aim: as a methods paper for forecast ensemble analysis. The manuscript proposes two potentially novel approaches to assessing individual forecast model contributions to overall ensemble performance: GAM-based regression to infer average model contributions to ensemble scores, and Pareto analysis to determine potentially optimal ensembles when there are multiple, possibly conflicting quantities to optimize when generating an ensemble. That said, I also think the paper would need to be expanded and re-framed to be a persuasive as a methods paper. In particular it would need in-depth analysis of the ensemble contribution inference methods (e.g. stress-testing the regression approach with different size sub-ensembles), as well as more detailed comparison with other candidate methods for assessing ensemble contributions such as those proposed in [3].

1.4 References to the manuscript text

In this review, all page references to the manuscript main text are to the page numbers in the compiled review PDF from Editorial Manager (including front-matter). All page references to the SI are to pages in the `.docx` file.

2 Principal Critiques

2.1 Insufficient detail on individual models limits the reader's ability to interpret key findings

The individual forecasting models the make up the ensemble are described only in a brief table in the supplement; the table itself is not referenced in the main text. This lack of information makes it much more difficult for the reader to assess or interpret key findings like "For pcWIS, we see that the GP growth rate model was detrimental to ensembles" (p. 13). This is the first reference to the GP growth rate model in the text. The statement a few sentences later that "The GP growth rate model is an experimental growth rate model, which can give unusual results when the growth rate is close to 0, which may explain poor performance, particularly around November to December" (p. 13) is difficult for the reader to assess given that they do not have key context about the model's assumptions, mathematical structure, why growth rates near zero might be problematic, or why the model was worth experimenting with.

R code implementing each of the models is available in the code repository accompanying the manuscript (example: `gam_rw` for influenza). These code files include model documentation written in the `roxygen2` documentation style (including mathematical equations). In my view, much or all of the content in that model documentation belongs at minimum in this manuscript's supplement, and some of it in the main text methods.

There are also a number of key configuration choices for the models used, including hyperparameter values, lengths of fitting windows, number of prior seasons considered when computing historical growth rates, etc. These choices also should be stated and then explained and justified within the manuscript itself.

If there are already published write-ups of the UKHSA's particular implementations / configurations of these models, those write-ups can be referenced in the main text or supplement in lieu of full model descriptions. Even then, though, a brief conceptual summary of each model is still needed.

2.2 Only a small subset of the forecasts and scores analyzed are visualized or otherwise reported

Only national level forecasts and corresponding observed data are plotted. I can imagine that data usage / privacy policies may prohibit visualizing forecasts and observed data for finer spatial scales (especially the ICB scale), but if so

this should be stated explicitly. If not, all regional forecasts and at least a representative subsample of ICB level forecasts should be visualized.

Simlarly, only national forecast scores are visualized. The scores for regional and ICB-level forecasts (both in real time and in the sub-ensemble retrospective analysis) should be reported. Breakdowns in score across time and space would also be valuable.

I appreciated the plots of the categorical predictions through time. To aid the reader in evaluating categorical forecast performance, it would be ideal to indicate the final observed category for each forecast date. It is not straightforward to read this off the plot of normalized incidence, since it requires the reader to eyeball a 20% increase or decrease.

It is not clear what the geographic granularity the average scores reported in supplement Table 3 correspond to. Are they the national scores only? Or are they are an average across all forecasts (regardless of spatial granularity)? If they are an average across all scores, it seems likely that they would mainly reflect the ICB-level forecasts (since there are many more of those and the per-capita scoring means that each individual forecast contributes to the average overall scores on the same scale. Why not have individual tables for national, regional, and ICB-level forecasts, rather than grouping them together?

Below, I give some illustrative examples of how the limited reporting and visualization of results makes it difficult to assess central manuscript claims.

2.2.1 Example: are operational ensembles indeed better than individual constituent models?

In the discussion, the authors claim "The operationally-delivered ensembles are usually better than individual models which is consistent with existing literature" (p. 17). The superiority or not of the ensemble to its individual constituent models is not analyzed in a systematic way in the text or figures of the paper. The closest we get to this comparison is the timeseries of probabilistic scores for operational ensemble (red) versus individual retrospective models (yellow) in for national forecasts in Figure 6. But that plot cannot fully support quoted statement above because:

1. The plot is crowded, with scores for the various sub-ensembles alongside the individual models and the operational ensemble. This makes it difficult to compare the individual models directly against the ensemble.

2. The plot only shows the national forecasts. The ensemble and the individual models each made forecasts (and were scored) at other spatial scales.

3. At least in some cases, there are sustained periods in the plot during which one or more individual models do outperform the operational ensemble

(e.g. for COVID-19 by pcWIS from mid-November through the pause in mid-December).

Without quantitative statements or clearer head-to-head visualizations it is hard to assess the claim that the ensemble was “usually better’.

2.2.2 Example: Why not construct full and/or paired retrospective ensembles?

It would be very useful context (and in particular help us understand what the retrospective analysis can and cannot tell us about operational individual model contributions operational ensemble) to know how well the retrospective models match the predictions and scores of the operational ensemble if (1) fully ensembled and/or (2) ensembled exactly as the real models were (i.e. shown in the bottom panels of Figure 1). This is also very important context for understanding the finding of retrospective sub-ensembles outperforming the operational ensemble. Are they outperforming it because they are better composed and omit detrimental models, or are they outperforming it because they are better forecasts on the whole?

2.2.3 Example: How consistent was operational performance across time and space?

The manuscript states that operational COVID-19 forecasts were generally good, with near theoretical empirical coverage overall. But it would be good to comment more on poor performance in the early going. Figure 1 suggests pretty consistent coverage misses for the first few weeks and consistent upward bias. What accounts for this? Does systematic overcoverage later in the season mask systematic undercoverage early by averaging out to normimal coverage? A plot of theoretical versus actual coverage over time would be of interest. This works particularly well at sub-national spatial scales, where it is possible to look at multiple sets of predictions and corresponding observations for each forecast date.

Similarly, more disaggregation of performance could help the reader assess the overall aggregate underconfidence of the ICB forecasts. Was calibration good at the level of individual ICBs? Was it good across ICBs throughout the season?

2.3 Key methodological choices are unclear or should be justified more

In a several places, I had questions about how exactly the analysis had been performed. In other places, it was clear what choice had been made, but I thought the choice needed a stronger justification.

2.3.1 Was vintaged data used for retrospective forecasts?

Retrospective counterfactual “forecasts” should ideally be produced using only data that would have been available to the hypothetical forecaster on the forecast date. It is not sufficient simply to exclude timeseries data (e.g. reported hopsitalizations) for dates after the forecast date. Instead, one should ideally use a “vintaged” or “as-of” version of the dataset as it would have appeared on that date, before any later revisions, anomaly corrections, or “backfill” altered timeseries data for the preceding days. Forecasts based on the ultimately reported (corrected, backfilled) data will tend to be better than those produced by a forecaster working with the data that is actually available to them in real time [2].

The manuscript should specify more details about how retrospective forecasting was conduced and discuss the lack of “as-of” datasets as a limitation when analyzing results. For example, this could be a key explanatory factor for “The (relatively) poor performance of our operational ensembles compared to retrospective ensembles” (p. 19).

Related: when scoring trend outcome, was the “realized value of the trend” based comparing the final rolling avg on the forecast date to the final rolling average 14 days later? (As opposed to, e.g. the value of the 14-day-rolling average on the forecast date as of the forecast date) I favor computing the realized value based on final data, but either way the method should be clarified.

2.3.2 Why use quantile-based scoring with a small number of quantiles when full posteriors are available?

If full posterior distributions were available per the methods, why score against a small number of predictive quantiles (via WIS) rather than computing the Continuous Ranked Probability Score (CRPS) based on all samples available? Were samples not archived in real time while quantiles were? What about retrospectively? If storage space constraints made it impossible to save all posterior samples from the retrospective analysis, sample-based scoring could have been performed within the model fitting loop before the posterior samples for a given ensemble were thrown away.

Given that forecasts were reported to stakeholders in quantile format, I could perhaps see an argument for using quantile-based scoring to evaluate all and only the predictive quantile ranges that were operationally reported to stakeholders. Here, additional (unreported) predictive quantile levels were used in scoring (but arguably as not as many as would be ideal for characterizing the full predictive distribution; most respiratory virus Forecast Hubs request more).

2.3.3 Why exclude RSV and norovirus?

Norovirus and RSV forecasts were produced in real time but excluded from the evaluation analysis and retrospective forecasting conducted in the manuscript.

It may be reasonable and justified to exclude RSV and norovirus, but the reader needs more context for the decision. This is especially true for RSV. The title refers to “respiratory forecasts”, so one potential justification for excluding norovirus is that it is typically considered a gastrointestinal rather than a respiratory pathogen. But then why exclude RSV?

The justification given in the text is “The basis of this paper will be to present and analyze forecasts of COVID-19 and Influenza hospital admissions, as the highest burden pathogens, with the most comprehensive ensembles” (p. 7). It is not clear what “most comprehensive” means here without additional context.

Later, in the future work section of the discussion, the authors write: “We will expand RSV and norovirus ensembles to gain the benefits which ensembles can bring to performance. This will allow us to perform similar analyses for those diseases” (p. 19). Were there simply not many models developed and used for RSV and norovirus? If so, it would be helpful to say something like “our ensemble modeling for RSV and norovirus was based on only 2 individual models, and so it is not amenable to the ensemble-contribution analysis we perform here”.

Even if the RSV and norovirus ensembles were based on fewer models, understanding how well those ensemble forecasts performed (compared to the larger influenza and COVID-19 ensembles) would be useful context for interpreting the results of the individual model ensemble contribution analysis.

2.3.4 What are the implications of scoring per capita incidence (versus log per capita incidence) for the interpretation of manuscript findings?

In explaining the decision to compute WIS for forecasts of per-capita incidence rather than count incidence, the manuscript cites [1]. A key point of that paper is that scoring raw positive-constrained epidemiological incidences (whether rescaled by population size or not) tends to weight performance during periods of high incidence heavily and be relatively insensitive to forecasting errors during periods of low incidence. Scoring a log-transformed incidence metric weights periods of different epidemiological incidence more equally. I would suggest that the authors should consider scoring $\log\left(\frac{\text{incidence}+1}{N}\right)$ or similar.

If they authors prefer scoring per-capita incidence to scoring log-transformed per-capita incidence, they should explain this choice and discuss the implications for their findings. This is particularly important for the interpretation of the Pareto analysis. The manuscript studies a potential conflict between optimizing predictions of daily incidence and optimizing predictions of categorical changes in the 7-day rolling average. As the authors of [1] point out, scoring a log-transformed incidence metric rewards accurate forecasts of the epidemic growth rate (closely related to the question of predicting changes in a 7-day rolling average incidence). Given this, some of the apparent conflict between goals might be mitigated if the target for incidence scoring were log-transformed.

2.3.5 Was individual model convergence assessed and if so how?

Were any automated convergence checks performed on the models in real time? In the retrospective analysis? The authors note "We see in the supplementary Figure A of retrospective forecasts that, on occasion, the GP growth rate forecasts exhibited unusual behaviour" (p. 15). I wonder whether these were symptoms of non-convergence.

3 Minor comments

3.1 Forecast pause

The absence of operational forecasts during the period of peak national influenza incidence is an unavoidable limitation of the real-time analysis, but it should be noted and discussed, particularly given that retrospective sub-ensembles and individual models performed poorly then (Figure 6, "Influenza forecasts were more challenging, especially when the peak was thought to be around the winter break in operations", p. 17). Note that the poor retrospetive peak performance is amplified by the decision to score per-capita incidence rather than log per capita incidence, but the RPS results suggest it is more general.

3.2 Why not compare the operational ensemble to its constituent individual operational models?

In the manuscript, the operational (real-time) ensemble gets compared to retrospective sub-ensembles and to retrospective individual models. The authors point out that it would not be a fair comparison to compare the overall aggregate score for the operational ensemble to overall aggregate scores for individual real-time models due to the fact that some individual real-time models were only used for a subset of forecasting problems. But elsewhere (e.g. Figure 6) the manuscript shows scores for the operational ensemble by forecast date. Why not compare the operational ensemble's scores for each date to its (real-time) constituent models from that date? Why not compare the operation ensemble overall to each of its constituent models on all and only the forecast problems where they overlap?

3.3 Figure 2 y-axis scale

A free y-axis for the scores plotted in Figure 2 is reasonable given that we do not necessarily expect the RPS and the pcWIS to have the same magnitudes, but I do not understand the motivation for using a log-scale y-axis for pcWIS but linear-scale y-axis for RPS. From supplement Figure D, it does not appear that there were ever predictions of the trend with complete certainty, so there should not be literal zero RPS values, and a log axis for that should be possible as well.

3.4 Figure 6: temporal resolution of scores

In Figure 6, score timeseries for individual models and sub-ensembles appear to have more frequent changepoints than the operational ensemble score (see e.g. the worst scoring individual model (highest yellow line) by log(pcWIS) for COVID-19 in mid-January, where we appear to have more-than-weekly scores (despite the scores being displayed "prediction start date", i.e. forecast date). Should this occur? If so, why?

3.5 Legend readability

Legend and axis text are very small for all figures. So are legend key glyphs. I strongly suggest making them larger to improve figure readability.

3.6 Wording suggestions

p. 9: "we define the effect $p_{m,l,t}$ of a component model m of ensemble r at time t and location level l". I would suggest rewording this a "we define the average effect $p_{m,l,t}$ of including a model m in an ensemble at time t and location level l". This rewording would avoid giving the impression that the effect is specific to some particular ensemble r in the way it is specific to a particular m, l, and t. In fact, the opposite is true: $p_{m,l,t}$ is an average over *all* ensembles that include m.

p. 13: "We see gradual upward trends in the performance of trend direction estimation for ETS, cubic regression and Gaussian process models." This is ambiguous. It could be read to imply model improvement ("upward trend in performance"). In fact, it appears to refer to the y-values in the heading upward over time. Given the meaningful of the y-axis, that in turn means *worsening* contributions to the ensemble (in some cases moving from helping to hurting).

p. 16: "That being said, our modal forecast was stable, so stakeholders received a correct forecast on the majority of weeks". It is not clear to me what this means. Is the point that just that for the majority of forecast weeks, the highest-predictive-probability category and the eventual observed value were both "stable"? Or is it a claim that there were weeks when RPS was not particular good (e.g. due to an uncertain forecast) but when the highest-predictive-probability category nonetheless ended up being to the observed category? Or something else?

References

[1] Nikos I Bosse et al. "Scoring epidemiological forecasts on transformed scales". In: *PLoS computational biology* 19.8 (2023), e1011393.

[2] Estee Y Cramer et al. "The United States COVID-19 forecast hub dataset". In: *Scientific data* 9.1 (2022), p. 462.

[3] Minsu Kim, Evan L Ray, and Nicholas G Reich. “Beyond forecast leaderboards: Measuring individual model importance based on contribution to ensemble accuracy”. In: *International Journal of Forecasting* (2026).
