# Supplementary material for: Evaluation of short-term multi-target respiratory forecasts over winter 2024-25 in England using sub-ensemble contribution analyses
Source: PLoS Comput Biol. 2026 Aug 12;22(8):e1014644. doi: 10.1371/journal.pcbi.1014644 (PMC13506087; doi:10.1371/journal.pcbi.1014644)
Supplement: Supplementary file 2 [file pcbi.1014644.s001.docx]

*S1 Fig. National norovirus case forecasts by prediction start date. Forecasts are for a 14-day horizon, with nowcasts not visualised. A single model was used rather than an ensemble. Norovirus cases increase over the season. Like other pathogens, there is a two-week break in forecasting over the Christmas period.*
