# Supplementary material for: Evaluation of short-term multi-target respiratory forecasts over winter 2024-25 in England using sub-ensemble contribution analyses
Source: PLoS Comput Biol. 2026 Aug 12;22(8):e1014644. doi: 10.1371/journal.pcbi.1014644 (PMC13506087; doi:10.1371/journal.pcbi.1014644)
Supplement: Supplementary file 3 [file pcbi.1014644.s002.docx]

*S2 Fig. RSV national operational forecasts (top) and model used (bottom). The first 6 RSV forecasts were not ensembles, they were single model forecasts (first a thin-plate spline, then a Gaussian process). The thin plate spline model was deprecated entirely early in the season due to beliefs about forecast credibility.*
