# Supplementary material for: Evaluation of short-term multi-target respiratory forecasts over winter 2024-25 in England using sub-ensemble contribution analyses
Source: PLoS Comput Biol. 2026 Aug 12;22(8):e1014644. doi: 10.1371/journal.pcbi.1014644 (PMC13506087; doi:10.1371/journal.pcbi.1014644)
Supplement: Supplementary file 4 [file pcbi.1014644.s003.docx]

*S3 Fig. RSV ensemble forecasts by NHS region.* *Black dots are daily admissions of RSV hospital admissions per day. Each region has similar disease dynamics: a peak in RSV admissions in early December, with a gradual decline in admissions. Incidence in each region peaks between 50 and 250 admissions.*
