# Supplementary material for: Evaluation of short-term multi-target respiratory forecasts over winter 2024-25 in England using sub-ensemble contribution analyses
Source: PLoS Comput Biol. 2026 Aug 12;22(8):e1014644. doi: 10.1371/journal.pcbi.1014644 (PMC13506087; doi:10.1371/journal.pcbi.1014644)
Supplement: Supplementary file 5 [file pcbi.1014644.s004.docx]

*S4 Fig. RSV operational ensemble admissions forecasts by age group (national geography). Daily admissions are shown as black points. Incidence varies greatly with each age group. The [0,2) group peaks in early December at just below 500 daily admissions, whereas the [65,75) age group peaks at around 70 daily admissions in late December. In general, younger age groups peak earlier than the older age groups.*
