# Supplementary material for: Evaluation of short-term multi-target respiratory forecasts over winter 2024-25 in England using sub-ensemble contribution analyses
Source: PLoS Comput Biol. 2026 Aug 12;22(8):e1014644. doi: 10.1371/journal.pcbi.1014644 (PMC13506087; doi:10.1371/journal.pcbi.1014644)
Supplement: Supplementary file 7 [file pcbi.1014644.s006.docx]

*S6 Fig. log(pcWIS) per prediction start date and NHS Region for RSV operational ensemble admissions forecasts. The performance of the ensemble is similar across regions, with each region showing increasing performance as we progress through the season. This improvement in performance could be attributed to (i) reduced incidence and (ii) improvements in the ensemble over the season.*
