# Supplementary material for: Evaluation of short-term multi-target respiratory forecasts over winter 2024-25 in England using sub-ensemble contribution analyses
Source: PLoS Comput Biol. 2026 Aug 12;22(8):e1014644. doi: 10.1371/journal.pcbi.1014644 (PMC13506087; doi:10.1371/journal.pcbi.1014644)
Supplement: Supplementary file 8 [file pcbi.1014644.s007.docx]

*S7 Fig. log(pcWIS) by age group (national geography) and prediction start date for RSV operational ensemble forecasts. Performance is generally worse for the very young, or very old age groups who typically have higher incidence. Within age groups, forecast performance tends to improve slightly as we progress through the season. This could be attributed to (i) reduced case counts (ii) development of the ensemble in real time*
