# Supplementary material for: Evaluation of short-term multi-target respiratory forecasts over winter 2024-25 in England using sub-ensemble contribution analyses
Source: PLoS Comput Biol. 2026 Aug 12;22(8):e1014644. doi: 10.1371/journal.pcbi.1014644 (PMC13506087; doi:10.1371/journal.pcbi.1014644)
Supplement: Supplementary file 9 [file pcbi.1014644.s008.docx]

*S8 Fig. log(RPS) by prediction start date for national single model Norovirus cases and national RSV operational ensemble forecasts. Norovirus scoring starts later in the season because (i) norovirus modelling started later in the season and (ii) trend direction estimation for norovirus was developed later in the season.*
