# Supplementary material for: Evaluation of short-term multi-target respiratory forecasts over winter 2024-25 in England using sub-ensemble contribution analyses
Source: PLoS Comput Biol. 2026 Aug 12;22(8):e1014644. doi: 10.1371/journal.pcbi.1014644 (PMC13506087; doi:10.1371/journal.pcbi.1014644)
Supplement: Supplementary file 10 [file pcbi.1014644.s009.docx]

*S9 Fig. log(RPS) by prediction start date and NHS Region for RSV operational ensemble admissions forecasts. Trend direction performance was similar across the season, with some early season estimates offering mixed results.*
