# Supplementary material for: Evaluation of short-term multi-target respiratory forecasts over winter 2024-25 in England using sub-ensemble contribution analyses
Source: PLoS Comput Biol. 2026 Aug 12;22(8):e1014644. doi: 10.1371/journal.pcbi.1014644 (PMC13506087; doi:10.1371/journal.pcbi.1014644)
Supplement: Supplementary file 11 [file pcbi.1014644.s010.docx]

*S10 Fig. log(RPS) by prediction start date and age group (national geography) for RSV operational ensemble admissions forecasts. Trend direction estimation performance was similar across the season, with some correct, and highly confident, forecasts early in the season in the two youngest age groups.*
