# Supplementary material for: Evaluation of short-term multi-target respiratory forecasts over winter 2024-25 in England using sub-ensemble contribution analyses
Source: PLoS Comput Biol. 2026 Aug 12;22(8):e1014644. doi: 10.1371/journal.pcbi.1014644 (PMC13506087; doi:10.1371/journal.pcbi.1014644)
Supplement: Supplementary file 12 [file pcbi.1014644.s011.docx]

*S11 Fig: (Top) A time series of admissions over time at the national level for COVID-19 and Influenza. The red line indicates the data UKHSA received initially, and in blue the final revised version of the data. (Bottom) The percentage change from the initially received admission count to the final counts over time for COVID-19 and Influenza. As national data is shown for simplicity, some local variation may be hidden due to cancelling directions. There are larger percentage changes for influenza at the start and end of the season where counts are low. The revisions for Influenza are largest near the winter holiday period, where operational forecasts were not reported.*
