# Supplementary material for: Evaluation of short-term multi-target respiratory forecasts over winter 2024-25 in England using sub-ensemble contribution analyses
Source: PLoS Comput Biol. 2026 Aug 12;22(8):e1014644. doi: 10.1371/journal.pcbi.1014644 (PMC13506087; doi:10.1371/journal.pcbi.1014644)
Supplement: Supplementary file 13 [file pcbi.1014644.s012.docx]

*S12 Fig: Map illustrating NHS regions and ICBs in England, and their approximate population sizes. The smallest geographies are coloured by their population sizes. The NHS regions and noted by black bold lines. London, due to its relatively small geographical area, is shown in a cut-out in the top right of the figure. Data processed and figure generated using R, using spatial boundaries and population catchment data. Source: Office for National Statistics licensed under the Open Government Licence v.3.0. Contains OS data Crown copyright and database right 2026. National boundaries:* [*https://geoportal.statistics.gov.uk/datasets/ons::countries-december-2024-boundaries-uk-buc-2/about*](https://geoportal.statistics.gov.uk/datasets/ons::countries-december-2024-boundaries-uk-buc-2/about) *; regional boundaries:* [*https://geoportal.statistics.gov.uk/datasets/ons::nhs-england-regions-january-2024-boundaries-en-bfc/about*](https://geoportal.statistics.gov.uk/datasets/ons::nhs-england-regions-january-2024-boundaries-en-bfc/about) *; ICB boundaries:* [*https://geoportal.statistics.gov.uk/datasets/ons::integrated-care-boards-april-2023-boundaries-en-bsc/about*](https://geoportal.statistics.gov.uk/datasets/ons::integrated-care-boards-april-2023-boundaries-en-bsc/about)
