# Supplementary material for: Evaluation of short-term multi-target respiratory forecasts over winter 2024-25 in England using sub-ensemble contribution analyses
Source: PLoS Comput Biol. 2026 Aug 12;22(8):e1014644. doi: 10.1371/journal.pcbi.1014644 (PMC13506087; doi:10.1371/journal.pcbi.1014644)
Supplement: Supplementary file 14 [file pcbi.1014644.s013.docx]

*S13 Fig: Diagnostic plots for COVID-19 pcWIS GAM. QQ plot shows moderate deviation from Normality. Histogram shows approximately symmetric residuals and suggests heavy-tailed residuals. Observed vs fitted values shows an approximately linear relationship, with a small cluster of outliers. The Residuals vs linear predictor shows three main groups (one per location level) and a small cluster of potential outliers. Overall, there is moderate deviation from Normality and homoscedasticity of residuals which may affect inferential statements, but predicted values are reasonably close to observed values. This is expected to have only a minor impact on pcWIS analysis.*
