# Supplementary material for: Evaluation of short-term multi-target respiratory forecasts over winter 2024-25 in England using sub-ensemble contribution analyses
Source: PLoS Comput Biol. 2026 Aug 12;22(8):e1014644. doi: 10.1371/journal.pcbi.1014644 (PMC13506087; doi:10.1371/journal.pcbi.1014644)
Supplement: Supplementary file 15 [file pcbi.1014644.s014.docx]

*S14 Fig: Diagnostic plots for COVID-19 RPS GAM. QQ plot shows moderate deviation from Normality. Histogram shows approximately symmetric residuals, there is mild assymetry and heavy-tailed residuals. Observed vs fitted values shows an approximately linear relationship, there is some mild curvature and moderate scatter. The Residuals vs linear predictor shows a decrease in residual variability as the value of the linear predictor increases, but no other obvious patterns. Overall, there is moderate deviation from Normality and homoscedasticity of residuals which may affect inferential and predictive statements. This could have a moderate effect on inferences of the COVID-19 RPS analysis.*
