# Supplementary material for: Evaluation of short-term multi-target respiratory forecasts over winter 2024-25 in England using sub-ensemble contribution analyses
Source: PLoS Comput Biol. 2026 Aug 12;22(8):e1014644. doi: 10.1371/journal.pcbi.1014644 (PMC13506087; doi:10.1371/journal.pcbi.1014644)
Supplement: Supplementary file 16 [file pcbi.1014644.s015.docx]

*S15 Fig: Diagnostic plots for Influenza pcWIS GAM. QQ plot shows moderate deviation from Normality. Histogram shows mildly asymmetric residuals. Observed vs fitted values shows an approximately linear relationship, with a small group of potential outliers when the fitted values are large. The Residuals vs linear predictor some evidence of residual variance increasing with the linear predictor. Overall, there is mild deviation from Normality and homoscedasticity of residuals which may affect inferential statements, but predicted values are reasonably close to observed values. This is expected to have only a minor impact on Influenza pcWIS analysis.*
