# Supplementary material for: Evaluation of short-term multi-target respiratory forecasts over winter 2024-25 in England using sub-ensemble contribution analyses
Source: PLoS Comput Biol. 2026 Aug 12;22(8):e1014644. doi: 10.1371/journal.pcbi.1014644 (PMC13506087; doi:10.1371/journal.pcbi.1014644)
Supplement: Supplementary file 17 [file pcbi.1014644.s016.docx]

*S16 Fig: Diagnostic plots for Influenza pcWIS RPS. QQ plot shows moderate deviation from Normality with one point having a very large, negative residual (approximate value, -6.5). Histogram shows mildly asymmetric residuals. Observed vs fitted values shows an approximately linear relationshipbut with variable scatter. The Residuals vs linear predictor some evidence of residual variance decreasing with the linear predictor. Overall, there is mild deviation from Normality and homoscedasticity of residuals which may affect inferential statements, but predicted values are reasonably close to observed values. The large potential outlier was for the gam_cr_gam_gp_ets sub-ensemble. This had a perfect RPS score of 0 which causes problems with a log transform. A small offset (*${10}^{-6})$ was added to all RPS values in the Influenza analysis to avoid $\log\left( 0 \right)$. However, the GAM still struggled to adequately account for this value. Overall, this is expected to have a moderate impact on Influenza pcWIS analysis.
