# Supplementary material for: Evaluation of short-term multi-target respiratory forecasts over winter 2024-25 in England using sub-ensemble contribution analyses
Source: PLoS Comput Biol. 2026 Aug 12;22(8):e1014644. doi: 10.1371/journal.pcbi.1014644 (PMC13506087; doi:10.1371/journal.pcbi.1014644)
Supplement: Supplementary file 18 [file pcbi.1014644.s017.docx]

*S17 Fig: Comparison of observed and nominal coverage statistics for forecasts by disease and spatial granularity, averaged over the 14 day prediction horizon. No bands are present for the national forecasts as there is only one national forecast per prediction start date-disease combination. The orange bands for Region and ICB represent the min and max value present across the locations. The blue, horizontal line represents the nominal coverage (90% or 50%, as appropriate).*
