# Supplementary material for: Evaluation of short-term multi-target respiratory forecasts over winter 2024-25 in England using sub-ensemble contribution analyses
Source: PLoS Comput Biol. 2026 Aug 12;22(8):e1014644. doi: 10.1371/journal.pcbi.1014644 (PMC13506087; doi:10.1371/journal.pcbi.1014644)
Supplement: Supplementary file 19 [file pcbi.1014644.s018.docx]

*S18 Fig: (Top) Trend direction probability estimates for national COVID-19 and Influenza admissions forecasts of the operational ensemble at national geography. (Bottom) Observed epidemic trend directions at national geography.*
