# Supplementary material for: Evaluation of short-term multi-target respiratory forecasts over winter 2024-25 in England using sub-ensemble contribution analyses
Source: PLoS Comput Biol. 2026 Aug 12;22(8):e1014644. doi: 10.1371/journal.pcbi.1014644 (PMC13506087; doi:10.1371/journal.pcbi.1014644)
Supplement: Supplementary file 20 [file pcbi.1014644.s019.docx]

*S19 Fig. Regional operational ensemble forecast for COVID-19 and Influenza. In these cases, the shapes of the admissions curves are similar to the corresponding national forecasts, but naturally, the number of admissions is lower at finer spatial resolutions.*
