# Supplementary material for: Evaluation of short-term multi-target respiratory forecasts over winter 2024-25 in England using sub-ensemble contribution analyses
Source: PLoS Comput Biol. 2026 Aug 12;22(8):e1014644. doi: 10.1371/journal.pcbi.1014644 (PMC13506087; doi:10.1371/journal.pcbi.1014644)
Supplement: Supplementary file 21 [file pcbi.1014644.s020.docx]

*S20 Fig: Example ICB forecasts for daily COVID-19 and Influenza hospital admissions. ICB names are redacted for data governance reasons. Like regional forecasts, ICB forecasts tend to follow a similar epidemic curve to the corresponding national forecast, but the scale of the epidemic is much smaller.*
