# Supplementary material for: Evaluation of short-term multi-target respiratory forecasts over winter 2024-25 in England using sub-ensemble contribution analyses
Source: PLoS Comput Biol. 2026 Aug 12;22(8):e1014644. doi: 10.1371/journal.pcbi.1014644 (PMC13506087; doi:10.1371/journal.pcbi.1014644)
Supplement: Supplementary file 22 [file pcbi.1014644.s021.docx]

*S21 Fig. log(pcWIS) and log(RPS) per disease-location level combination. Note for some individual models, the trend direction estimate was near perfect, so the log scores diverge to* $-\infty$ *and are therefore not visible on the plot.*
