# Supplementary material for: Evaluation of short-term multi-target respiratory forecasts over winter 2024-25 in England using sub-ensemble contribution analyses
Source: PLoS Comput Biol. 2026 Aug 12;22(8):e1014644. doi: 10.1371/journal.pcbi.1014644 (PMC13506087; doi:10.1371/journal.pcbi.1014644)
Supplement: Supplementary file 23 [file pcbi.1014644.s022.docx]

**Retrospective forecasts**

*S22 Fig. Individual retrospective model predictions for COVID-19 hospital admission at national geography. The nation wave during the study period included a decline then stable period with limited variation.*
