# Supplementary material for: Evaluation of short-term multi-target respiratory forecasts over winter 2024-25 in England using sub-ensemble contribution analyses
Source: PLoS Comput Biol. 2026 Aug 12;22(8):e1014644. doi: 10.1371/journal.pcbi.1014644 (PMC13506087; doi:10.1371/journal.pcbi.1014644)
Supplement: Supplementary file 24 [file pcbi.1014644.s023.docx]

*S23 Fig. Individual retrospective model predictions for Influenza hospital admissions at national geography. The epidemic wave showed a sharp rise in admissions followed by a more gradual decline.*
