# Supplementary material for: Evaluation of short-term multi-target respiratory forecasts over winter 2024-25 in England using sub-ensemble contribution analyses
Source: PLoS Comput Biol. 2026 Aug 12;22(8):e1014644. doi: 10.1371/journal.pcbi.1014644 (PMC13506087; doi:10.1371/journal.pcbi.1014644)
Supplement: Supplementary file 25 [file pcbi.1014644.s024.docx]

*S24 Fig. (Top) Bubble chart showing log(RPS) vs log(pcWIS) for different forecasting locations, point size represents the population of the location for forecasting. (Middle) Same bubble chart with an outlier removed; there is an outlier ICB for COVID-19 with a log(pcWIS) value of approximately -3.5. Upon further investigation, we saw the final week of data for this ICB reported zero admissions every day. It is unclear if this is a poor forecast or a data quality issue, as admissions counts in the very late season are often very low (below 5 admissions per day).) Boxplot showing population sizes per location level. In general, scores are lower (better) for locations with a larger population. (Bottom) Boxplot showing population sizes per location level.*
