# Supplementary material for: Evaluation of short-term multi-target respiratory forecasts over winter 2024-25 in England using sub-ensemble contribution analyses
Source: PLoS Comput Biol. 2026 Aug 12;22(8):e1014644. doi: 10.1371/journal.pcbi.1014644 (PMC13506087; doi:10.1371/journal.pcbi.1014644)
Supplement: Supplementary file 27 [file pcbi.1014644.s026.docx]

| **Model Type** | **Disease** | **Score** | **Model** | **Nation** | **Region** | **ICB** |
| --- | --- | --- | --- | --- | --- | --- |
| **COVID-19** |  |  |  |  |  |  |
| Individual Models | COVID-19 | pcWIS | All individual models | $4.61 \times{10}^{-7}$ | $5.99 \times{10}^{-7}$ | $1.52 \times{10}^{-3}$ |
| Matched Ensemble | COVID-19 | pcWIS | Matched Ensemble | $3.40 \times{10}^{-7}$ | $5.26 \times{10}^{-7}$ | $\boldsymbol{1.11 \times}\boldsymbol{10}^{\boldsymbol{-6}}$ |
| Operational Ensemble | COVID-19 | pcWIS | Operational Ensemble | $4.69 \times{10}^{-7}$ | $5.94 \times{10}^{-7}$ | $9.84 \times{10}^{-4}$ |
| Retrospective Model | COVID-19 | pcWIS | ets | $6.98 \times{10}^{-7}$ | $7.75 \times{10}^{-7}$ | $9.30 \times{10}^{-4}$ |
| Retrospective Model | COVID-19 | pcWIS | gam_cr | $5.05 \times{10}^{-7}$ | $6.00 \times{10}^{-7}$ | $1.70 \times{10}^{-3}$ |
| Retrospective Model | COVID-19 | pcWIS | gam_gp | $5.12 \times{10}^{-7}$ | $6.31 \times{10}^{-7}$ | $1.70 \times{10}^{-3}$ |
| Retrospective Model | COVID-19 | pcWIS | gam_rw | $\boldsymbol{2.33 \times}\boldsymbol{10}^{\boldsymbol{-7}}$ | $\boldsymbol{4.25 \times}\boldsymbol{10}^{\boldsymbol{-7}}$ | $2.38 \times{10}^{-3}$ |
| Retrospective Model | COVID-19 | pcWIS | gr_gp | $4.35 \times{10}^{-7}$ | $6.11 \times{10}^{-7}$ | $9.24 \times{10}^{-4}$ |
| Retrospective Model | COVID-19 | pcWIS | gr_mean | $2.17 \times{10}^{-7}$ | $4.59 \times{10}^{-7}$ | $1.47 \times{10}^{-3}$ |
| Individual Models | COVID-19 | RPS | All individual models | $1.88 \times{10}^{-1}$ | $4.47 \times{10}^{-1}$ | $5.37 \times{10}^{-1}$ |
| Matched Ensemble | COVID-19 | RPS | Matched Ensemble | $9.50 \times{10}^{-2}$ | $2.93 \times{10}^{-1}$ | $3.96 \times{10}^{-1}$ |
| Operational Ensemble | COVID-19 | RPS | Operational Ensemble | $1.63 \times{10}^{-1}$ | $3.78 \times{10}^{-1}$ | $4.85 \times{10}^{-1}$ |
| Retrospective Model | COVID-19 | RPS | ets | $1.95 \times{10}^{-1}$ | $4.93 \times{10}^{-1}$ | $5.66 \times{10}^{-1}$ |
| Retrospective Model | COVID-19 | RPS | gam_cr | $2.40 \times{10}^{-1}$ | $4.11 \times{10}^{-1}$ | $4.81 \times{10}^{-1}$ |
| Retrospective Model | COVID-19 | RPS | gam_gp | $1.56 \times{10}^{-1}$ | $4.73 \times{10}^{-1}$ | $5.07 \times{10}^{-1}$ |
| Retrospective Model | COVID-19 | RPS | gam_rw | $\boldsymbol{8.17 \times}\boldsymbol{10}^{\boldsymbol{-2}}$ | $3.38 \times{10}^{-1}$ | $4.65 \times{10}^{-1}$ |
| Retrospective Model | COVID-19 | RPS | gr_gp | $3.09 \times{10}^{-1}$ | $5.21 \times{10}^{-1}$ | $6.20 \times{10}^{-1}$ |
| Retrospective Model | COVID-19 | RPS | gr_mean | $9.95 \times{10}^{-2}$ | $4.32 \times{10}^{-1}$ | $6.38 \times{10}^{-1}$ |
| **Influenza** |  |  |  |  |  |  |
| Individual Models | Influenza | pcWIS | All individual models | $1.34 \times{10}^{-6}$ | $1.54 \times{10}^{-6}$ | $2.10 \times{10}^{-6}$ |
| Matched Ensemble | Influenza | pcWIS | Matched Ensemble | $6.75 \times{10}^{-7}$ | $9.37 \times{10}^{-7}$ | $1.41 \times{10}^{-6}$ |
| Operational Ensemble | Influenza | pcWIS | Operational Ensemble | $5.62 \times{10}^{-7}$ | $\boldsymbol{8.55 \times}\boldsymbol{10}^{\boldsymbol{-7}}$ | $1.47 \times{10}^{-6}$ |
| Retrospective Model | Influenza | pcWIS | ets | $1.05 \times{10}^{-6}$ | $1.27 \times{10}^{-6}$ | $1.93 \times{10}^{-6}$ |
| Retrospective Model | Influenza | pcWIS | gam_cr | $2.49 \times{10}^{-6}$ | $2.27 \times{10}^{-6}$ | $2.50 \times{10}^{-6}$ |
| Retrospective Model | Influenza | pcWIS | gam_gp | $1.22 \times{10}^{-6}$ | $1.63 \times{10}^{-6}$ | $2.37 \times{10}^{-6}$ |
| Retrospective Model | Influenza | pcWIS | gam_rw | $7.98 \times{10}^{-7}$ | $1.19 \times{10}^{-6}$ | $1.84 \times{10}^{-6}$ |
| Retrospective Model | Influenza | pcWIS | historic_gr_median | $\boldsymbol{2.81 \times}\boldsymbol{10}^{\boldsymbol{-7}}$ | $6.28 \times{10}^{-7}$ | $1.07 \times{10}^{-6}$ |
| Individual Models | Influenza | RPS | All individual models | $2.71 \times{10}^{-1}$ | $4.15 \times{10}^{-1}$ | $4.51 \times{10}^{-1}$ |
| Matched Ensemble | Influenza | RPS | Matched Ensemble | $\boldsymbol{1.60 \times}\boldsymbol{10}^{\boldsymbol{-1}}$ | $\boldsymbol{2.45 \times}\boldsymbol{10}^{\boldsymbol{-1}}$ | $\boldsymbol{3.04 \times}\boldsymbol{10}^{\boldsymbol{-1}}$ |
| Operational Ensemble | Influenza | RPS | Operational Ensemble | $1.61 \times{10}^{-1}$ | $2.88 \times{10}^{-1}$ | $3.55 \times{10}^{-1}$ |
| Retrospective Model | Influenza | RPS | ets | $2.72 \times{10}^{-1}$ | $4.20 \times{10}^{-1}$ | $4.93 \times{10}^{-1}$ |
| Retrospective Model | Influenza | RPS | gam_cr | $3.21 \times{10}^{-1}$ | $4.67 \times{10}^{-1}$ | $4.66 \times{10}^{-1}$ |
| Retrospective Model | Influenza | RPS | gam_gp | $2.60 \times{10}^{-1}$ | $3.89 \times{10}^{-1}$ | $4.28 \times{10}^{-1}$ |
| Retrospective Model | Influenza | RPS | gam_rw | $2.44 \times{10}^{-1}$ | $3.91 \times{10}^{-1}$ | $4.19 \times{10}^{-1}$ |
| Retrospective Model | Influenza | RPS | historic_gr_median | $2.03 \times{10}^{-1}$ | $3.65 \times{10}^{-1}$ | $4.15 \times{10}^{-1}$ |

S1 Table. Mean scores for models used in winter operations. Smaller values indicate better forecasts. In all cases, the average ensemble score for each location level-disease-score group was less than the average operational individual model. The best model per disease-score-location level combination is highlighted in bold.
