# Supplementary material for: Evaluation of short-term multi-target respiratory forecasts over winter 2024-25 in England using sub-ensemble contribution analyses
Source: PLoS Comput Biol. 2026 Aug 12;22(8):e1014644. doi: 10.1371/journal.pcbi.1014644 (PMC13506087; doi:10.1371/journal.pcbi.1014644)
Supplement: Supplementary file 28 [file pcbi.1014644.s027.docx]

| **Disease** | **Retrospective Sub-ensemble members of Pareto Front** |
| --- | --- |
| COVID-19 | gam_cr, ets, gr_mean |
|  | gam_cr, gam_gp, gam_rw |
|  | gam_cr, gam_gp, gr_mean |
|  | gam_cr, gam_rw, gr_mean |
|  | gam_gp, ets, gr_mean |
|  | gam_gp, gam_rw, gr_mean |
|  | gam_rw, ets, gr_mean |
| Influenza | gam_cr, ets, historic_gr_median |
|  | gam_cr, gam_rw, historic_gr_median |
|  | gam_rw, ets, historic_gr_median |
|  | gam_rw, gam_gp, historic_gr_median |

*S2 Table. Members of Pareto Front (retrospective sub-ensembles) for full Pareto analysis (not using weighted averages) by disease.* *Of the 20 retrospective sub-ensembles per disease, 7 sit on the Pareto Front for COVID-19, and 4 sit on the Pareto Front for Influenza.*
