# Supplementary material for: Evaluation of short-term multi-target respiratory forecasts over winter 2024-25 in England using sub-ensemble contribution analyses
Source: PLoS Comput Biol. 2026 Aug 12;22(8):e1014644. doi: 10.1371/journal.pcbi.1014644 (PMC13506087; doi:10.1371/journal.pcbi.1014644)
Supplement: Supplementary file 29 [file pcbi.1014644.s028.docx]

| ***Disease*** | ***Spatial granularity*** | ***Observed 90% coverage*** | ***Observed 50% coverage*** |
| --- | --- | --- | --- |
| *COVID-19* | *Nation* | *87.5%* | *56.3%* |
|  | *Region* | *91.2%* | *53.7%* |
|  | *ICB* | *95.2%* | *65.5%* |
| *Influenza* | *Nation* | *90.6%* | *62.0%* |
|  | *Region* | *91.8%* | *59.9%* |
|  | *ICB* | *94.6%* | *71.0%* |

S3 Table. Comparison of nominal coverage of prediction intervals to observed coverage by disease and spatial granularity for operational ensembles. Statistics are an average for the entire season and where relevant, averaged across locations.
