# Supplementary material for: Evaluation of short-term multi-target respiratory forecasts over winter 2024-25 in England using sub-ensemble contribution analyses
Source: PLoS Comput Biol. 2026 Aug 12;22(8):e1014644. doi: 10.1371/journal.pcbi.1014644 (PMC13506087; doi:10.1371/journal.pcbi.1014644)
Supplement: Supplementary file 30 [file pcbi.1014644.s029.docx]

| **Disease** | **Metric** | **Minimum** | **Overall mean** | **Maximum** | **Operational mean** |
| --- | --- | --- | --- | --- | --- |
| COVID-19 | pcWIS | $1.52\times{10}^{-7}$ | $1.73\times{10}^{-6}$ | $2.02\times{10}^{-7}$ | $4.69\times{10}^{-7}$ |
| COVID-19 | RPS | $3.86\times{10}^{-2}$ | $0.0531\times{10}^{-2}$ | $0.0729\times{10}^{-2}$ | $1.88\times{10}^{-1}$ |
| Influenza | pcWIS | $6.69\times{10}^{-7}$ | $1.06\times{10}^{-6}$ | $1.31\times{10}^{-6}$ | $5.62\times{10}^{-7}$ |
| Influenza | RPS | $1.64\times{10}^{-1}$ | $1.90\times{10}^{-1}$ | $2.14\times{10}^{-1}$ | $2.71\times{10}^{-1}$ |

*S4 Table. Minimum, overall mean, and maximum values of retrospective ensemble scores averaged across the season for national predictions; with operational ensemble average for comparison.*
