# Supplementary material for: Evaluation of short-term multi-target respiratory forecasts over winter 2024-25 in England using sub-ensemble contribution analyses
Source: PLoS Comput Biol. 2026 Aug 12;22(8):e1014644. doi: 10.1371/journal.pcbi.1014644 (PMC13506087; doi:10.1371/journal.pcbi.1014644)
Supplement: Supplementary file 31 [file pcbi.1014644.s030.docx]

| **Model type** | **Location level** | **Age group granularity** | **Disease** | **Score name** | **Mean score** |
| --- | --- | --- | --- | --- | --- |
| **Norovirus** |  |  |  |  |  |
| Single operational model | Nation | None | Norovirus | pcWIS | $2.20 \times{10}^{1}$ |
| Single operational model | Nation | None | Norovirus | RPS | $5.88 \times{10}^{-1}$ |
| **RSV** |  |  |  |  |  |
| Operational ensemble | Nation | None | RSV | pcWIS | $1.04 \times{10}^{-6}$ |
| Operational ensemble | Nation | Fine | RSV | pcWIS | $5.98 \times{10}^{-6}$ |
| Operational ensemble | Region | None | RSV | pcWIS | $1.55 \times{10}^{-6}$ |
| Operational ensemble | Nation | None | RSV | RPS | $4.56 \times{10}^{-1}$ |
| Operational ensemble | Nation | Fine | RSV | RPS | $5.83 \times{10}^{-1}$ |
| Operational ensemble | Region | None | RSV | RPS | $5.01 \times{10}^{-1}$ |

*S5* *Table. Mean scores for models used in winter operations. Smaller values indicate better forecasts. In all cases.*
