# Supplementary material for: Evaluation of short-term multi-target respiratory forecasts over winter 2024-25 in England using sub-ensemble contribution analyses
Source: PLoS Comput Biol. 2026 Aug 12;22(8):e1014644. doi: 10.1371/journal.pcbi.1014644 (PMC13506087; doi:10.1371/journal.pcbi.1014644)
Supplement: Supplementary file 32 [file pcbi.1014644.s031.docx]

# S1 Section

# RSV admissions and Norovirus cases analysis

RSV admissions are forecast at the regional level, whilst also accounting for the age group of the admitted patient. Forecasts are then aggregated to one of three levels for reporting (and evaluation) purposes:

- National forecast (all ages)
- National forecast (by age group)
- Regional forecast (all ages)

The regions are the same NHS regions as for COVID-19 and Influenza forecasts. Age groups are [0, 2), [2, 5), [5, 18), [18, 65), [65, 76), and [75, 120) years old. These groups were selected with the help of epidemiologists with expertise in RSV and winter pathogens.

Norovirus cases are a joint nowcast and forecast model see (cite) for details. In the 24/25 season, norovirus modelling was done only at the national scale and the forecast was a single model. By the 25/26 season this was developed into a regional ensemble model. We also note that storage of norovirus results started later in the season compared to other pathogens as it was a new forecasting target for the 24/25 season.
