# Supplementary material for: Evaluation of short-term multi-target respiratory forecasts over winter 2024-25 in England using sub-ensemble contribution analyses
Source: PLoS Comput Biol. 2026 Aug 12;22(8):e1014644. doi: 10.1371/journal.pcbi.1014644 (PMC13506087; doi:10.1371/journal.pcbi.1014644)
Supplement: Supplementary file 33 [file pcbi.1014644.s032.docx]

# S2 Section

A specification of the novel models added in the 2024-2025 season for COVID-19 and Influenza forecasts. Hyperparameters are provided within the configuration files in <https://github.com/jcken95/sub-ensemble-evaluation>.

GP growth rate model (COVID-19)

At a high level the aim of this model was to estimate a crude instantaneous growth rate from hospital admissions, then forecast the GR forward in time using a Gaussian process. Using this forecasted growth rate and recent values of admissions, future admissions could be simulated.

At a high level the model:

- calculates a local growth rate based on rolling averages of the target

- estimate this local growth rate with a short training length and Gaussian process model in time

- apply this forecasted growth rate forward in time to the most recent target.

Throughout we will refer to the growth rate as $GR_{t}=\log\left( s\left( \frac{Y_{t}}{Y_{t-1}} \right) \right)$

Where $s\left( \right)$ is some smoothing function - in our case a seven day rolling mean and $Y_{1}, Y_{2}\ldots Y_{t}$ is our admissions time series.

We then define our regression as

$$\mathrm{GR}_{t} = \mathrm{icb} + GP_{\mathrm{region}}\left( t \right)+ GP_{\mathrm{nation}}\left( t \right)$$

$\mathrm{icb}$ is a random effect for the ICB, $\mathrm{GP}_{location level}$ is a Gaussian process smooth pooled at the given location level. Once the $\mathrm{GR}$ has been estimated we need to convert back to admissions.

We take the forecasted growth rate up to $\mathrm{GR}_{t_{\max}+14}.$ The smooth estimates of admissions $s\left( Y_{t_{\max}} \right)$ are taken which can be extrapolated from.

The forecast for $t>t_{max}$ is therefore the summed growth rate up to $t$.

$$s\left( Y_{t} \right)=s\left( Y_{t_{\max}} \right)\times e^{\sum\mathrm{GR}_{t}}$$

However, this gives the smoothed admissions, so we can incorporate uncertainty according to a $\mathrm{NegBin}$.

$$Y_{t} \sim\mathrm{NegBin}\left( s\left( y_{t}), \theta\right) \right)$$

The model is tuned primarily by:

- how much data is fed into the model (training length)

- varying GP hyperparameters

- how long the rolling averages on the model smoothers are.

- dispersion parameter $\theta$.

Mean GR model (COVID-19)

The premise of this model is similar to that of “GP growth rate”, however, instead of a Gaussian process to estimate the time varying instantaneous growth, we estimate a short term fixed growth rate for each ICB. This fixed growth rate is then assumed to continue forward in time. Similarly, the future growth rate is then applied to recent admissions values to simulate future growth.

At a high level the model:

- calculates a local growth rate based on rolling averages of the target

- estimate this local growth rate with a short training length and linear model in time

- apply this forecasted growth rate forward in time to the most recent target.

Throughout we will refer to the growth rate as $GR_{t}=\log\left( s\left( \frac{Y_{t}}{Y_{t-1}} \right) \right)$

Where $s\left( \right)$ is some smoothing function - in our case a seven day rolling mean and $Y_{1}, Y_{2}\ldots Y_{t}$ is our admissions time series.

We then define our regression as

$$\mathrm{GR}_{t} = \mathrm{icb}_{\mathrm{region}}$$

$\mathrm{icb}_{\mathrm{region}}$ is a nested random effect for the ICB in each region. Once the $\mathrm{GR}$ has been estimated we need to convert back to admissions.

We take the forecasted growth rate up to $\mathrm{GR}_{t_{\max}+14}.$ The smooth estimates of admissions $s\left( Y_{t_{\max}} \right)$ are taken which can be extrapolated from.

The forecast for $t>t_{max}$ is therefore the summed growth rate up to $t$.

$$s\left( Y_{t} \right)=s\left( Y_{t_{\max}} \right)\times e^{\sum\mathrm{GR}_{t}}$$

However, this gives smoothed admissions, so we can incorporate uncertainty according to a $\mathrm{NegBin}$.

$$Y_{t} \sim\mathrm{NegBin}\left( s\left( y_{t}), \theta\right) \right)$$

The model is tuned primarily by:

- how much data is fed into the model (training length)

- how long the rolling averages on the model smoothers are.

- dispersion parameter $\theta$.

Median GR model (Influenza)

Conceptually the aim for this model is to leverage, in a straightforward way, past trends of influenza admissions. Using the SARI-Watch surveillance system, a weekly growth rate for each of the past seasons is calculated. While Influenza is highly heterogeneous each season, there are some generalized patterns each year, particularly in terms of direction rather than magnitude. A median growth rate from past seasons is estimated. Matching the current admissions data to past epiweeks we know the next two weeks’ median growth rate, which is applied to the current admissions data to produce a forecast. To generate uncertainty and day-of-week effects, we fit a random walk model to the data assuming a national trend, then apply the growth rate correction to this random walk forecast.

The core assumption is that there is information in past season growth rates that can help predict this season. This would be particularly helpful to capture early and late season dynamics, as well as behavioral changes that occur due to times of year (such as holiday periods).

The assumption is not about absolute values, but rather relative changes over time. Past seasons are matched to the current one by epidemic week `lubridate::epiweek`. To incorporate this trend we use a GAM RW model first, of the form:

$$Y_{t}\sim\mathrm{NegBin}\left( \mu_{t}, \theta\right)$$

$$\log E\left( Y_{t} \right)= \mathrm{do}w_{t} +\mathrm{ic}b_{\mathrm{region}} + W_{t} +\log\left( \mathrm{population} \right)$$

Where $W_{t}$ is a random walk; if $Z_{\tau}\sim N\left( 0, \sigma\right)$ are i.i.d. random variables then:

$W_{t}=\sum_{\tau=1}^{t} Z_{\tau}$ is a Gaussian random walk.

$Y_{t}$ is the number of admissions at time $t$, $\mathrm{do}w_{t}$ is a fixed effect for the day of week, $\mathrm{ic}b_{\mathrm{region}}$ is a random effect by ICB, nested within regions and $\log\left( \mathrm{population} \right)$serves as an offset term.

With this model, we take the past observed growth rates (in this case, the median of previous seasons). We accumulate the historic daily growth rate $gr_{t}$ over the prediction horizon as $GR_{t}$. For $t\geq prediction start date, Y_{t}=Y_{t}*GR_{t}$. Which gives a multiplicative factor based on past data and a random walk.

For the 24/25 season, the following past Influenza seasons were used to estimate the median: 2016-17, 2017-2018, 2018-2019, 2022-2023, 2023-2024. These years represent a mixture of pre-and-post COVID-19 public health social measures years.
