# Supplementary material for: Evaluation of short-term multi-target respiratory forecasts over winter 2024-25 in England using sub-ensemble contribution analyses
Source: PLoS Comput Biol. 2026 Aug 12;22(8):e1014644. doi: 10.1371/journal.pcbi.1014644 (PMC13506087; doi:10.1371/journal.pcbi.1014644)
Supplement: Supplementary file 34 [file pcbi.1014644.s033.docx]

**Review for PCOMPBIOL-D-26-00338:** Evaluation of short-term multi-target respiratory forecasts over winter 2024-25 in England using sub-ensemble contribution analyses

I enjoyed the manuscript and think that the work represents an important step forward in evaluating probabilistic forecasts. Below are a couple general comments followed by pointed comments by section. I look forward to reading the revised manuscript.

**General comments:**

Throughout the manuscript the reference to “ensemble” and “sub-ensemble” was confusing. A sub-ensemble as a group of three models. But sometimes “ensemble” refers to one of these groups of three models, a random group of models used during operational forecasts, or a stack of posterior predictions. The manuscript would be greatly improved if these terms were edited to only reference one idea (i.e. “group” or “cluster” for the 3-model ensembles).

I like the PF analysis, and the idea of “Optimizing performance for a single metric represents an oversimplification of practical forecasting challenges and diverse customer needs” (from Methods) is the thread that ties the manuscript together. If this idea can be expanded upon in the introduction/discussion the manuscript would appeal to a larger audience.

**Abstract**

The term sub-ensemble is undefined in the Methods subsection.

COVID-10 should be COVID-19 in the Results subsection.

The absolute scores in the Results subsection will be meaningless to readers who are unfamiliar with proper scoring rules, perhaps sticking to the relative metrics here will increase readability.

UKHSA is undefined in the Interpretation subsection.

**Introduction**

The phrase “individual models’ contributions to sub-ensembles” should be clarified – hard to know what this means without reading the Methods section first.

The authors might consider adding precision (to go along with accuracy) in the last sentence, as proper scoring rules take both metrics into account.

**Methods**

For the non-UK reader, it would be nice to see a map of how ICBs sit within regions, and how the regions sit within the UK.

Was the sample size of admissions the same across the spatial hierarchy? If not, how do you think this affects your results and interpretation? Were models (or sub-ensembles) more skillful in highly populated areas?

Can the authors expand on what “An upward change after the end of a 14-day period in the 7-day rolling average of the forecasting target” means? The forecast is for 14 days into the future, but the target is the 7-day rolling average at the end of the 14 days? So, the trend in the first seven days of the forecast doesn’t matter?

The 20% cutoff for ordinal classifications seems arbitrary without justification, can the authors provide a justification/citation for this cutoff?

The sentence “The relative frequency of each event across posterior samples gives us a trend direction probability” is confusing. Can the authors elaborate?

The equation for $pcIS_{\alpha}$ should be a displayed equation and labeled as equation 1. What is currently labeled as equation 1 is the 5^th^ displayed equation on the text. Additionally, $\alpha, l, u$ need definitions in the text.

Some clarifications about the GAMs. How many knots were used for each smoother? The ensemble subscript *r* refers to a specific group of three models? How well does the GAM predict the observed data? There is no analysis (in the main text or supplementary material) that evaluates the validity of the GAM.

Can the authors expand on how they dealt with forecast horizon? The GAM ignores forecast horizon but equation 1 says “over a fixed forecast horizon.” So, what is this fixed amount? Also, the mention of a smoother “over all forecast horizons” implies that there are scores for all 14 days of the forecast. If that is true, how do authors think ignoring this affects their results? If scores decline with forecast horizon, did some models perform better at earlier horizons and others at late horizons?

At the end of the methods section the authors state “A “full” Pareto analysis which uses all scores at all spatial geographies is performed with results available in the supplementary materials (Table 3).” The referenced table is missing. What is currently labeled as Table 3 in the supplementary material is not what this text refers to.

Table 1 in the supplementary material should be in the main text.

**Results**

All figure captions need to be updated. The authors rely on the main text to describe the figures instead of having a caption that can be used to interpret the figures as standalone products.

Results might be easier to interpret if scores are log transformed as they are in figure 2.

Minimum mean, overall mean, and maximum mean need to be defined. These reference the correct supplementary table 3.

**Discussion**

How do authors think the choice of using groups of three for sub-ensembles influenced their results? Would the same models proven most useful in the PF analysis stay that way if groups of two were used? What about four?

The weights chosen for the PF analysis make sense from a national perspective, but I would think that practitioners on the ground would weigh ICBs or regions more. How do the authors think reweighting would affect their results? No need to rerun the analysis, but I do think this is an interesting discussion point given the choice to weigh national forecasts as twice as important as ICB and regional forecasts. Moreover, as aggregation occurs forecasts are likely to become more skillful. So again, do the authors think that down weighting the (most likely) less skillful forecasts affected their results? Perhaps some models are better at the national scale and others at the ICB?

The authors state “this resulted in all of our modelling outputs being delivered on time, or ahead of schedule without failure.” But there was a gap in the forecasts over winter break. Can the authors clarify this? Another discussion point could be how to develop the cyberinfrastructure so that the break in forecasts does not occur, especially since that was during peak Influenza.

LASOMO and LOMO need to be defined.
