# Supplementary material for: Evaluation of short-term multi-target respiratory forecasts over winter 2024-25 in England using sub-ensemble contribution analyses
Source: PLoS Comput Biol. 2026 Aug 12;22(8):e1014644. doi: 10.1371/journal.pcbi.1014644 (PMC13506087; doi:10.1371/journal.pcbi.1014644)
Supplement: Supplementary file 35 [file pcbi.1014644.s036.docx]

# Reviewer Response “Evaluation of short-term multi-target respiratory forecasts over winter 2024-25 in England using sub-ensemble contribution analyses”

We thank the editor and reviewers for their careful reading and engagement with our work. We found the feedback extremely helpful and agree the manuscript is much improved by incorporating their suggestions. In almost all cases we have followed the suggested revisions exactly. We have added considerably more explanation in the manuscript regarding methods and results. There are substantially more visualisations in the paper & supplement which we feel fleshes out the work more and better represents the research we have conducted. Reviewer comments are presented below in black with our point-by-point responses in red.

## Editor’s comments

Both reviewers found the manuscript interesting and provided helpful guidance for improving it. However, for further consideration of publication, substantially more details are needed for understanding methods and results. There are also some methodological issues raised by reviewers that will likely require re-analysis and additional visuals.

Currently the manuscript is framed as methods development but fails to describe what application of this method has revealed in terms of the biology of the system. Adding some text describing the novel biological insights will be important in revision. i.e., What is learned from knowing which models were useful at different epidemic stages? What additional information is gained from using this method over previous forecasting models for this system? The authors mention the approach allows for faster decision-making. What are the impacts?

We agree that these factors could be more clearly stated in the manuscript and have added the following paragraph to the discussion to draw out the points mentioned:

“This research has shown that the complex biological systems of infections over time are difficult to forecast consistently well, with varying model performance over time – shown by the GAM based contribution analysis. Furthermore, we showed that different aspects of the system – absolute scores vs relative change are not equally easy to forecast under each model, with different combinations of models having different skill, as demonstrated by the Pareto Front analysis. We showed that models which depend on growth rate assumptions tend to add value in incline and decline phases, whereas models that assume time series trends or flat forecasts can improve performance when epidemic waves are more stochastically variable. Having the tools available to inspect different aspects of ensemble performance allows public health forecasters to evaluate more rigorously, and therefore draw better conclusions to plan future model combinations.”

## Reviewer 1

I enjoyed the manuscript and think that the work represents an important step forward in evaluating probabilistic forecasts. Below are a couple general comments followed by pointed comments by section. I look forward to reading the revised manuscript.

**General comments:**

Throughout the manuscript the reference to “ensemble” and “sub-ensemble” was confusing. A sub-ensemble as a group of three models. But sometimes “ensemble” refers to one of these groups of three models, a random group of models used during operational forecasts, or a stack of posterior predictions. The manuscript would be greatly improved if these terms were edited to only reference one idea (i.e. “group” or “cluster” for the 3-model ensembles).

We agree with the reviewer that the wording used initially in this manuscript was confusing and did not clarify what type of ensemble was being referred to at different times – conflating sub-ensembles with the concept of an ensemble. We have checked for every reference of “ensemble” in the manuscript to ensure they are clearly noted as “operational ensemble”, “sub-ensemble” or “retrospective ensemble”, with all definitions given in text.

I like the PF analysis, and the idea of “Optimizing performance for a single metric represents an oversimplification of practical forecasting challenges and diverse customer needs” (from Methods) is the thread that ties the manuscript together. If this idea can be expanded upon in the introduction/discussion the manuscript would appeal to a larger audience.

We are glad to have the opportunity to expand upon this idea from the manuscript in the main text. We have more clearly articulated our user types in the introduction:

“For the winter 2024-25 season, UKHSA developed a suite of short-term forecast models for COVID-19, Influenza, RSV and norovirus. These forecasts were delivered widely within the health system in England. Users of UKHSA’s forecasts included:

- Senior officials and politicians in central government (DHSC) responsible for policy.
- National and local public health officials responsible for interventions, resourcing and surveillance.
- National healthcare system managers responsible for resilience, supply chains and system integration.
- Regional and local healthcare system managers responsible for capacity, bed and staff management in primary/secondary care.”

We have expanded on the description of our forecast users, and furthermore added the below to the introduction:

“A common challenge in evaluating epidemic forecasts for practitioners in public health is the choice of evaluation scoring rule. Often a variety of rules are presented in research, but often only a single evaluation metric is used to guide model development. The choice of which evaluation metric to optimize against is challenging as different users have different needs. For example, a hospital capacity manager may be primarily interested in local absolute values of hospital admissions, whereas a national policy maker may care more about the national direction of an epidemic. Designing a model that is only good for one user type, but poor for another is unsatisfying and an area for further work. In this work we tackle the competing priorities in epidemic forecast evaluation using a Pareto front analysis.”

And to the discussion:

“When discussing requirements with users of our forecasts there was no clear consensus on what geography or scale (relative or absolute) the forecasts were most important for. This is because the purpose of the forecast varies for the different audience members. For some users the accuracy in terms of absolute values was critical, whereas others found the general direction most important and for many a mix is useful. Users of the forecasts have responsibilities across the national, regional and local scales. These competing priorities are challenging to directly encode, which motivated our use of a Pareto front analysis. This analysis enables us to highlight the trade-off between different models directly on the front, making the choice between performance for different user types more explicit. The Pareto front gives us a much broader view on the model choice problem than our GAM-based averaging approach on a single evaluation metric alone. An alternative approach to this problem, would be to encode multivariate scoring rules, which should be explored operationally as future work (cite).

**Abstract**

The term sub-ensemble is undefined in the Methods subsection.

COVID-10 should be COVID-19 in the Results subsection.

The absolute scores in the Results subsection will be meaningless to readers who are unfamiliar with proper scoring rules, perhaps sticking to the relative metrics here will increase readability.

UKHSA is undefined in the Interpretation subsection.

We thank the reviewer for the suggested edits to the abstract, which they have caught on our behalf. We have implemented all three of them all which improved the readability of the section.

**Introduction**

The phrase “individual models’ contributions to sub-ensembles” should be clarified – hard to know what this means without reading the Methods section first.

The authors might consider adding precision (to go along with accuracy) in the last sentence, as proper scoring rules take both metrics into account.

To introduce the sub-ensemble analysis more clearly, we have reworded this section taking the reviewers comments onboard to:

“In this work we explore sub-ensembles, an ensemble formed from a subset of available models, to help draw more general conclusions about individual contributing models. We aim to understand how individual models contribute to forecast accuracy & precision by evaluating a range of possible ensembles to compare their performance. We then explore how the individual model’s contribution changes over time and geography to help inform future seasons ensemble composition.”

**Methods**

For the non-UK reader, it would be nice to see a map of how ICBs sit within regions, and how the regions sit within the UK.

We have added Figure L to the supplementary material. This illustrates the ICBs, NHS regions and gives an approximate population size per ICB.

Was the sample size of admissions the same across the spatial hierarchy? If not, how do you think this affects your results and interpretation? Were models (or sub-ensembles) more skillful in highly populated areas?

We agree that population sizes do have an impact on forecastability, and have produced a plot to compare across the different location levels. For a given spatial granularity, population sizes in a location level are similar in magnitude. Figure X in the supplementary material shows how model skill (measured by scores) changes with population size. Broadly, when the population is larger, forecasts have lower pcWIS and RPS scores. The figure also contains a boxplot showing population sizes per region. We have added the following text below figure 2:

“To compare the relative skill of forecasts at different population levels we refer the reader to Figure X. pcWIS and RPS are both scale-agnostic scoring rules, however, we see that, in general, larger population areas generally correspond to lower (better) score values. This suggests that the larger geographies are usually easier to predict.”

Can the authors expand on what “An upward change after the end of a 14-day period in the 7-day rolling average of the forecasting target” means? The forecast is for 14 days into the future, but the target is the 7-day rolling average at the end of the 14 days? So, the trend in the first seven days of the forecast doesn’t matter?

We agree with the reviewer that this could be clearer for the reader, and have expanded the description to the following:

“Our secondary forecast target approach is an epidemic trend direction assessment. We categorise an epidemic as one of “decreasing”, “stable”, or “increasing”, an ordinal classification. This is a simplification of our forecasts, but useful for many users. The trend direction for a given forecast day is calculated by first calculating the 7-day right aligned rolling average of forecast target and forecast sample value. The direction is then a percentage change between the rolling average target, and a forecast 14 days ahead (our forecast horizon). A change between target data and forecast value of ≥20% is considered an “increase”, while a downward change of ≥20% is a “decrease”. Changes less than 20% are considered “stable”. This procedure produces trend directions for each time step in the forecast horizon, up to the final forecast day. For forecast users we only show the final forecast trend direction as the most relevant overall indication of direction. This method has desirable qualities – we can use the approach across many pathogens without alteration and can deploy it at a range of spatial and population scales, which are more challenging with absolute changes.”

The 20% cutoff for ordinal classifications seems arbitrary without justification, can the authors provide a justification/citation for this cutoff?

This is an important point and not something we have publicly articulated previously. While all thresholds are arbitrary this method has been agreed with users and has a justification on the basis of other user-tested analysis. We have added the below:

“The specific threshold of 20% was chosen following consultation with forecast users as a meaningful change in trend that maintained coherence with other surveillance metrics users were familiar with. Surveillance indicators within UKHSA and elsewhere are often classified using a 10% week-on-week change, which we extend to our 2-week horizon, which would be a 21% increase (1.1×1.1=1.21). We approximate the 21% change to 20% to make the method clearer for users. We are currently considering how to extend this approach to even longer time horizons, but feedback from users will be the primary driver.”

The sentence “The relative frequency of each event across posterior samples gives us a trend direction probability” is confusing. Can the authors elaborate?

We have reworded the sentence to:

“We calculate the trend direction probability as the proportion of posterior samples that between each threshold boundary, for example if 350 of 500 posterior sample predictions meet the “increase” definition, we have a probability of increase of 70%.”

The equation for

$$pcIS_{\alpha}$$

 should be a displayed equation and labeled as equation 1. What is currently labeled as equation 1 is the 5^th^ displayed equation on the text. Additionally,

$$\alpha, l, u$$

need definitions in the text.

We agree that this could have been clearer to the reader. All equations have been (re)numbered. Explicit definitions of $\alpha, l, u$ have now been given just before the definition of $pcIS_{\alpha}$

Some clarifications about the GAMs. How many knots were used for each smoother? The ensemble subscript *r* refers to a specific group of three models? How well does the GAM predict the observed data? There is no analysis (in the main text or supplementary material) that evaluates the validity of the GAM.

The number of knots is chosen so that a knot is places every two weeks. This is now explained in the main manuscript (alongside additional details of the smoother) just below equation 4:

“for each smooth we used penalised thin plate regression splines with knots placed every two weeks”

Yes, $r$ refers to a group of three models (a sub-ensemble). We have clarified the language and terminology around this just below equation 4:

“where $S_{r,l}\left( t \right)$ is the average score of sub-ensemble $r$for a location level $l$ (i.e. nation, region, ICB)”

Figures M-P of the supplementary material now give some diagnostics of the GAMs, with captions describing the validity of the GAMs. They show some issues with the fitted GAMs – mostly surrounding outliers. The reader is directed to the GAMs with the directly above Equation 5

“We use standard residual-based diagnostics to investigate our GAMs with results in figures M-P of the supplementary information. There are some minor to moderate issues with our GAMs which are a limitation of the analyses relying on the GAMs.”

We have also added a comment about the use of GAMs in our limitations section:

“The GAMs fit to scores for Figures 3 and 4 to understand how individual models contribute to sub-ensemble performance, like all models, have their limitations. Diagnostic plots for the GAMs are given in Figures M-P. Future work should explore strength and limitations of the used of statistical model-based ensemble analysis.”

Can the authors expand on how they dealt with forecast horizon? The GAM ignores forecast horizon but equation 1 (now Eq 5) says “over a fixed forecast horizon.” So, what is this fixed amount? Also, the mention of a smoother “over all forecast horizons” implies that there are scores for all 14 days of the forecast. If that is true, how do authors think ignoring this affects their results? If scores decline with forecast horizon, did some models perform better at earlier horizons and others at late horizons?

Thank you for highlighting this issue around our explanation. We included the following definition below equation 5

“Where $S_{r,l}\left( t \right)$ is the mean score of the forecasts across the forecast horizon (days 1 to 14)”

At the end of the methods section the authors state “A “full” Pareto analysis which uses all scores at all spatial geographies is performed with results available in the supplementary materials (Table 3).” The referenced table is missing. What is currently labeled as Table 3 in the supplementary material is not what this text refers to.

We apologise for the misdirection. We have now referred the reader to Supplementary Table 2, which does contain the results of the full Pareto analysis. This is referred to just above Figure 1:

“A “full” Pareto analysis which uses all scores at all spatial geographies is performed with results available in the supplementary materials (Supplementary Table 2).”

Table 1 in the supplementary material should be in the main text.

We have now moved the table containing the model descriptions into the main text to make it easier for the reader to understand the intuition of each model. Furthermore, we have added more details to the table (including hierarchical assumptions, which we feel is relevant to the later analysis), and added the following paragraph for context:

“The models used operationally in the 2024-25 season are described in Table 1. Each GAM model is a permutation of previous work ([cite](https://www.nature.com/articles/s43856-023-00424-4)), with the ETS models also described in ([cite](https://academic.oup.com/ije/article/54/3/dyaf066/8156944#522508837)). The GP growth rate, Mean GR and Median GR models were conceptually new to the English ensemble this year. Full implementation details are available in the supporting code <https://github.com/jcken95/sub-ensemble-evaluation>.”

**Results**

All figure captions need to be updated. The authors rely on the main text to describe the figures instead of having a caption that can be used to interpret the figures as standalone products.

Results might be easier to interpret if scores are log transformed as they are in figure 2.

Minimum mean, overall mean, and maximum mean need to be defined. These reference the correct supplementary table 3.

We have implemented all three suggestions above and agree that this improves the interpretability of the results.

**Discussion**

How do authors think the choice of using groups of three for sub-ensembles influenced their results? Would the same models proven most useful in the PF analysis stay that way if groups of two were used? What about four?

This is a really interesting question raised which we have tried to answer. Ideally, this would be an experiment within itself, we have given our thoughts in the discussion, copied below:

“We selected a model combination size of 3 models for the sub-ensemble analysis to produce generate sufficient combinations of models to perform this analysis and have meaningful ensembles. The performance of a given sub-ensemble is a combination of 1) the accuracy & precision of each individual model and 2) the interaction between individual models when ensembled, such as whether model properties such as bias and sharpness cancel. As a result, there should be some similarity between individual models on the Pareto front if the sub-ensemble size were to change. However, the variance of scores across sub-ensembles would decrease as the sub-ensemble size increases. A larger cohort of models would be needed to explore this effect in future work.”

The weights chosen for the PF analysis make sense from a national perspective, but I would think that practitioners on the ground would weigh ICBs or regions more. How do the authors think reweighting would affect their results? No need to rerun the analysis, but I do think this is an interesting discussion point given the choice to weigh national forecasts as twice as important as ICB and regional forecasts. Moreover, as aggregation occurs forecasts are likely to become more skillful. So again, do the authors think that down weighting the (most likely) less skillful forecasts affected their results? Perhaps some models are better at the national scale and others at the ICB?

We thank the reviewer for this feedback – it’s a point we had discussed when writing the manuscript but had not clearly articulated in the final version. We have added in the below paragraph in the discussion:

“The geographical weights chosen for the weighted Pareto front analysis are challenging to select and represent our view of the average utility across all users of each spatial level of forecasting. A different opinion of the relative utility of each spatial level may produce a different Pareto front of sub-ensembles. While each user group would prefer higher weighting of the geography most relevant to their work, we assume that the national forecast is used more often by local users than visa-versa to understand overall pressure as well as their own. However, due to aggregation of forecasts and different model structures, not all models perform equally well across spatial scales. Some models assume a national trend in admissions, whereas others assume each ICB is independent which would further drive the possible variation in results due to a different weighting scheme.”

The authors state “this resulted in all of our modelling outputs being delivered on time, or ahead of schedule without failure.” But there was a gap in the forecasts over winter break. Can the authors clarify this? Another discussion point could be how to develop the cyberinfrastructure so that the break in forecasts does not occur, especially since that was during peak Influenza.

We thank the reviewer for highlighting, and we have added further context to the paragraph with the below additional text:

“Notable however, is the Christmas winter holiday period, where there is an agreed pause with users due to changes in surveillance reporting cadences, staff availability and senior officials working. This year, this pause occurred during the peak of the influenza wave, a period of high-pressure meaning users relied on older forecasts to make decisions. While automation of modelling can improve delivery, capacity of staff trained to assure work is also a key challenge, and forecasts must align to other surveillance data cadences they rely on. The pause occurred during a time of heightened pressure, and when the influenza wave peaked – a period particularly challenging to forecast. The difficulty in this period is highlighted by the retrospective models poor performance during this time shown in Figure 6, across both pcWIS and RPS.”

LASOMO and LOMO need to be defined.

We apologize for this oversight, and have now provided definitions.

## Reviewer 2

**1.3 Alternative possible framing**

I have assessed the manuscript principally as it does or does not achieve the three scientific aims I enumerated above. That said, there is another potential interpretation of the manuscript’s scientific aim: as a methods paper for forecast ensemble analysis. The manuscript proposes two potentially novel approaches to assessing individual forecast model contributions to overall ensemble performance: GAM-based regression to infer average model contributions to ensemble scores, and Pareto analysis to determine potentially optimal ensembles when there are multiple, possibly conflicting quantities to optimize when generating an ensemble. That said, I also think the paper would need to be expanded and re-framed to be a persuasive as a methods paper. In particular it would need in-depth analysis of the ensemble contribution inference methods (e.g. stress testing the regression approach with different size sub-ensembles), as well as more detailed comparison with other candidate methods for assessing ensemble contributions such as those proposed in [3].

We thank the reviewer for this useful feedback. It reflects a feeling that we had during the investigation and writing of this paper, that it has multiple purposes. Our primary objective for the work was to report the operational ensemble and explore how we could have delivered a better one, with the secondary aim being to introduce these methods we explored in trying to answer this question. We hope that our revision has helped to provide assurance for the methods presented, but we agree further investigation of the methods presented could be a valuable academic contribution for future work.

**2 Principal Critiques**

**2.1 Insufficient detail on individual models limits the reader’s ability to interpret key findings**

The individual forecasting models the make up the ensemble are described only in a brief table in the supplement; the table itself is not referenced in the main text. This lack of information makes it much more difficult for the reader to assess or interpret key findings like ”For pcWIS, we see that the GP growth rate model was detrimental to ensembles” (p. 13). This is the first reference to the GP growth rate model in the text. The statement a few sentences later that “The GP growth rate model is an experimental growth rate model, which can give unusual results when the growth rate is close to 0, which may explain poor performance, particularly around November to December” (p. 13) is difficult for the reader to assess given that they do not have key context about the model’s assumptions, mathematical structure, why growth rates near zero might be problematic, or why the model was worth experimenting with.

This was a really helpful suggestion, and we have implemented it in full, with the suggestions below. We have now added Table 1, which gives an intuition for each model discussed in the paper. Furthermore, in the supplement we have a full model specification for the GP growth rate, in addition to the code already provided.

R code implementing each of the models is available in the code repository accompanying the manuscript (example: gam rw for influenza). These code files include model documentation written in the roxygen2 documentation style (including mathematical equations). In my view, much or all of the content in that model documentation belongs at minimum in this manuscript’s supplement, and some of it in the main text methods.

There are also a number of key configuration choices for the models used, including hyperparameter values, lengths of fitting windows, number of prior seasons considered when computing historical growth rates, etc. These choices also should be stated and then explained and justified within the manuscript itself.

If there are already published write-ups of the UKHSA’s particular implementations / configurations of these models, those write-ups can be referenced in the main text or supplement in lieu of full model descriptions. Even then, though, a brief conceptual summary of each model is still needed.

We thank the reviewer for this feedback. We agree that the individual models used in this work could be better described to help the reader understand our work better. We have added in Table 1, which outlines the intuition behind each model, and key dirstructures. Furthermore, we have referenced our past works where full structural details are provided, and added Supplementary Section B for the new models added in this season. We would highlight, however, that this research is focused heavily on ensemble contributions, and analysis – for which full implementation of all hyperparameters may be beyond of scope for understanding the key messages of the manuscript. In comparable ensemble analysis research (DOIs: [10.7554/eLife.81916](https://doi.org/10.7554/eLife.81916), [10.1073/pnas.2113561119](https://doi.org/10.1073/pnas.2113561119), 10.1038/s41467-024-50601-9, [10.1371/journal.pcbi.1014199](https://doi.org/10.1371/journal.pcbi.1014199)) full model details are not given in text, and only in some cases are the code provided as we have done.

**2.2 Only a small subset of the forecasts and scores analyzed are visualized or otherwise reported**

Only national level forecasts and corresponding observed data are plotted. I can imagine that data usage / privacy policies may prohibit visualizing forecasts and observed data for finer spatial scales (especially the ICB scale), but if so this should be stated explicitly. If not, all regional forecasts and at least a representative subsample of ICB level forecasts should be visualized.

We agree that sub-national forecasts may be of interest to the reader. Figures S and T have been added to the supplementary information to display all regional predictions (Figure S) from the operational ensembles per disease and a subset of 4 ICB level forecasts (Figure T) from operational ensembles. A subset of ICB locations have been presented with location names anonymised for data governance purposes, which is also mentioned in the figure captions. There is a reference to the figures just below figure 1 in the main text:

 “Regional forecasts, and a collection of ICB forecasts are available in Figures S and T.”

Similarly, only national forecast scores are visualized. The scores for regional and ICB-level forecasts (both in real time and in the sub-ensemble retrospective analysis) should be reported. Breakdowns in score across time and space would also be valuable.

We have updated Figure 2 to show scores for the operational ensembles and models used to construct operational ensembles. We have also added Supplementary Figure U and Supplementary Table 3 to show scores over time over different spatial resolutions for models used in our winter operations. We have also updated the abstract (results) to indicate the improvement in performance operational ensembles offer over individual models.

I appreciated the plots of the categorical predictions through time. To aid the reader in evaluating categorical forecast performance, it would be ideal to indicate the final observed category for each forecast date. It is not straightforward to read this off the plot of normalized incidence, since it requires the reader to eyeball a 20% increase or decrease.

We thank the reviewer for highlighting this and we agree that adding the final observed category helps the reader interpret the results more clearly. We have updated Figure R of the supplementary material to show the observed epidemic trend (and updated the caption accordingly).

It is not clear what the geographic granularity the average scores reported in supplement Table 3 correspond to. Are they the national scores only? Or are they are an average across all forecasts (regardless of spatial granularity)? If they are an average across all scores, it seems likely that they would mainly reflect the ICB-level forecasts (since there are many more of those and the per-capita scoring means that each individual forecast contributes to the average overall scores on the same scale. Why not have individual tables for national, regional, and ICB-level forecasts, rather than grouping them together?

We apologise for the oversight, these are for national forecasts only. We have revised the table and figure captions to make geographies clear when the figure or table does not directly reference the geography.

Below, I give some illustrative examples of how the limited reporting and visualization of results makes it difficult to assess central manuscript claims.

**2.2.1 Example: are operational ensembles indeed better than individual constituent models?**

In the discussion, the authors claim “The operationally-delivered ensembles are usually better than individual models which is consistent with existing literature” (p. 17). The superiority or not of the ensemble to its individual constituent models is not analyzed in a systematic way in the text or figures of the paper. The closest we get to this comparison is the timeseries of probabilistic scores for operational ensemble (red) versus individual retrospective models (yellow) in for national forecasts in Figure 6. But that plot cannot fully support quoted statement above because:

1. The plot is crowded, with scores for the various sub-ensembles alongside the individual models and the operational ensemble. This makes it difficult to compare the individual models directly against the ensemble.

2. The plot only shows the national forecasts. The ensemble and the individual models each made forecasts (and were scored) at other spatial scales.

3. At least in some cases, there are sustained periods in the plot during which one or more individual models do outperform the operational ensemble (e.g. for COVID-19 by pcWIS from mid-November through the pause in mid-December).

Without quantitative statements or clearer head-to-head visualizations it is hard to assess the claim that the ensemble was “usually better’.

We have added Supplementary Table 1 and Figure U to show how – within season – the operational ensembles perform relative to individual real-time models. These are referenced in the main text

“Comparisons of performance over time for the operational ensembles against individual models at each spatial resolution are given in Figure U; the operational ensemble is rarely the best model but typically outperforms most individual models. Season average scores for operational ensembles and individual models are given in Supplementary Table 1 for COVID-19 and Influenza; this shows that the ensemble was on average, provided both better numerical forecasts and trend direction forecasts of COVID-19 and Influenza at all spatial resolutions.”

The following explicitly addresses points 1-3:

1. We have included Figure U to explicitly show individual performance vs operational ensemble performance.

2. Figure U shows model performance across different spatial scales. Table 1 now includes information about season average scores per spatial scale.

3. Numerical scores (Supplementary Table 1) that on average, the operational ensemble was best. The plots (Figure U) also show that the operational ensemble usually outperforms the individual models.

**2.2.2 Example: Why not construct full and/or paired retrospective ensembles?**

It would be very useful context (and in particular help us understand what the retrospective analysis can and cannot tell us about operational individual model contributions operational ensemble) to know how well the retrospective models match the predictions and scores of the operational ensemble if (1) fully ensembled and/or (2) ensembled exactly as the real models were (i.e. shown in the bottom panels of Figure 1). This is also very important context for understanding the finding of retrospective sub-ensembles outperforming the operational ensemble. Are they outperforming it because they are better composed and omit detrimental models, or are they outperforming it because they are better forecasts on the whole?

This is an interesting point. We have addressed this by including a “matched ensemble” to Figure 6. This ensemble is constructed using retrospective forecasts, but uses the same combination of models at the operational ensemble, i.e. the second of the above suggestions. This gives us an equivalent forecast. The matched ensemble usually outperforms the operational ensemble. This suggests a slight bias in favour of the retrospective models. We have added the following text just below Figure 6:

“We also include a “matched ensemble”. This is an ensemble of the individual retrospective models, where the individual models used to construct the ensemble are the same as the models in the operational ensemble. The matched ensembles typically, but not strictly, outperformed the operational ensembles; the matched ensemble was especially good for Influenza. This suggests a bias in favour of retrospectively fitted models and sub-ensembles over the operational ensemble as they are fit using revised data and end-of-season hyperparameters.”

**2.2.3 Example: How consistent was operational performance across time and space?**

The manuscript states that operational COVID-19 forecasts were generally good, with near theoretical empirical coverage overall. But it would be good to comment more on poor performance in the early going. Figure 1 suggests pretty consistent coverage misses for the first few weeks and consistent upward bias. What accounts for this? Does systematic overcoverage later in the season mask systematic undercoverage early by averaging out to normimal coverage? A plot of theoretical versus actual coverage over time would be of interest. This works particularly well at sub-national spatial scales, where it is possible to look at multiple sets of predictions and corresponding observations for each forecast date.

Similarly, more disaggregation of performance could help the reader assess the overall aggregate underconfidence of the ICB forecasts. Was calibration good at the level of individual ICBs? Was it good across ICBs throughout the season?

This is a really helpful suggestion from the reviewer, which we have fully implemented. We agree that a better understanding of coverage over time and across spatial scales helps the reader understand the model performance better. We have added Figure Q to the supplementary material which plots nominal coverage against observed for each disease-location level combination. We have also added the following text just below Figure 1 in the main manuscript

“Figure Q of the supplementary material shows that the nominal 90% prediction intervals often had close to 90% coverage. For COVID-19 the forecasts were most closely calibrated after the winter break. For Influenza, the nominal coverage was usually greater than expected, with a drop in coverage in mid November. There were unusual reporting patterns at this time of year corresponding to a minor influenza outbreak”

**2.3 Key methodological choices are unclear or should be justified more**

In a several places, I had questions about how exactly the analysis had been performed. In other places, it was clear what choice had been made, but I thought the choice needed a stronger justification.

**2.3.1 Was vintaged data used for retrospective forecasts?**

Retrospective counterfactual “forecasts” should ideally be produced using only data that would have been available to the hypothetical forecaster on the forecast date. It is not sufficient simply to exclude timeseries data (e.g. reported hopsitalizations) for dates after the forecast date. Instead, one should ideally use a “vintaged” or “as-of” version of the dataset as it would have appeared on that date, before any later revisions, anomaly corrections, or “backfill” altered timeseries data for the preceding days. Forecasts based on the ultimately reported (corrected, backfilled) data will tend to be better than those produced by a forecaster working with the data that is actually available to them in real time [2].

The manuscript should specify more details about how retrospective forecasting was conduced and discuss the lack of “as-of” datasets as a limitation when analyzing results. For example, this could be a key explanatory factor for “The (relatively) poor performance of our operational ensembles compared to retrospective ensembles” (p. 19).

Related: when scoring trend outcome, was the “realized value of the trend” based comparing the final rolling avg on the forecast date to the final rolling average 14 days later? (As opposed to, e.g. the value of the 14-day-rolling average on the forecast date as of the forecast date) I favor computing the realized value based on final data, but either way the method should be clarified.

We thank the reviewer for highlighting this opportunity to improve the manuscript. Backfilling of forecast targets is naturally a substantial challenge to handle in many settings. The data UKHSA uses to forecast is rapid management information obtained directly from England’s unified healthcare system – which is quite unique. Because of this collection, there are limited revisions of the target. As a result for this data in real-time we effectively treat it as “complete”, without correction. We do however, of course agree that articulating our assumptions and justifying them is important. As a result we have added a Supplementary Figure K which highlights the scale of the revisions present, and added further context to the manuscript to address the details proposed.

We have added the following to the description of the forecast target:

“The data can be revised for up to 14 days following submission, however, in practice the extent of revisions is small shown in Supplementary Figure K.”

In addition, we have added the following line in the evaluation section of the methods:

“In all cases we evaluate the predictions against the finalised data set collected after all revisions have been received (14 days following the end of the data forecasted).”

In the description of the retrospective experiment, we have also added the following:

“The models and data used to generate these forecasts are based on the final model configurations at the end of the season, and revised data. However, the scale of data revisions is small for this data, with an average 1.3% and 4% change for COVID-19 and Influenza respectively, shown in Supplementary Figure K.”

And, in the Limitations section we have explicitly stated that we expect revisions to play a role in the comparative performance:

“Furthermore, data is revised in real-time which can degrade model performance operationally when compared to models forecast using retrospectively available data.”

**2.3.2 Why use quantile-based scoring with a small number of quantiles when full posteriors are available?**

If full posterior distributions were available per the methods, why score against a small number of predictive quantiles (via WIS) rather than computing the Continuous Ranked Probability Score (CRPS) based on all samples available? Were samples not archived in real time while quantiles were? What about retrospectively? If storage space constraints made it impossible to save all posterior samples from the retrospective analysis, sample-based scoring could have been performed within the model fitting loop before the posterior samples for a given ensemble were thrown away. Given that forecasts were reported to stakeholders in quantile format, I could perhaps see an argument for using quantile-based scoring to evaluate all and only the predictive quantile ranges that were operationally reported to stakeholders.

Here, additional (unreported) predictive quantile levels were used in scoring (but arguably as not as many as would be ideal for characterizing the full predictive distribution; most respiratory virus Forecast Hubs request more).

We agree with the reviewer that our original approach was inconsistent and could be improved. We have selected a WIS-like metric to be consistent with much of the wider literature for epidemic forecasting, and agree with the reviewer’s suggestion that we only evaluate quantiles presented to users, and have therefore revised the manuscript to use the median, 50% and 90% intervals. The other quantiles were present as an artifact of stakeholder preference due to familiarity of using 66% and 95% intervals frequently seen in other statistical analyses, which we have now removed. We have assumed that even with low numbers of quantiles the WIS is a strong proxy for CRPS, which would be a possible future work to validate.

**2.3.3 Why exclude RSV and norovirus?**

Norovirus and RSV forecasts were produced in real time but excluded from the evaluation analysis and retrospective forecasting conducted in the manuscript. It may be reasonable and justified to exclude RSV and norovirus, but the reader needs more context for the decision. This is especially true for RSV. The title refers to “respiratory forecasts”, so one potential justification for excluding norovirus is that it is typically considered a gastrointestinal rather than a respiratory pathogen. But then why exclude RSV?

The justification given in the text is “The basis of this paper will be to present and analyze forecasts of COVID-19 and Influenza hospital admissions, as the highest burden pathogens, with the most comprehensive ensembles” (p. 7). It is not clear what “most comprehensive” means here without additional context. Later, in the future work section of the discussion, the authors write: “We will expand RSV and norovirus ensembles to gain the benefits which ensembles can bring to performance. This will allow us to perform similar analyses for those diseases” (p. 19). Were there simply not many models developed and used for RSV and norovirus? If so, it would be helpful to say something like “our ensemble modeling for RSV and norovirus was based on only 2 individual models, and so it is not amenable to the ensemble-contribution analysis we perform here”.

Even if the RSV and norovirus ensembles were based on fewer models, understanding how well those ensemble forecasts performed (compared to the larger influenza and COVID-19 ensembles) would be useful context for interpreting the results of the individual model ensemble contribution analysis.

This suggestion addresses an inconsistency in our paper, which we now have the opportunity to rectify. We have updated the relevant methods section with:

“The basis of this paper is primarily to present and analyse forecasts of COVID-19 and Influenza hospital admissions with the most comprehensive ensembles. Both COVID-19 & Influenza ensembles contain more than three models, whereas RSV contained only three models, and norovirus a single model. For this reason, we present and evaluate the RSV and norovirus forecasts in Supplementary Figures A-J. We exclude these pathogens from the retrospective sub-ensemble analysis due to having insufficient operational models developed this season.”

**2.3.4 What are the implications of scoring per capita incidence (versus log per capita incidence) for the interpretation of manuscript findings?**

In explaining the decision to compute WIS for forecasts of per-capita incidence rather than count incidence, the manuscript cites [1]. A key point of that paper is that scoring raw positive-constrained epidemiological incidences (whether rescaled by population size or not) tends to weight performance during periods of high incidence heavily and be relatively insensitive to forecasting errors during periods of low incidence. Scoring a log-transformed incidence metric weights periods of different epidemiological incidence more equally. I would suggest that the authors should consider scoring

If they authors prefer scoring per-capita incidence to scoring log-transformed per-capita incidence, they should explain this choice and discuss the implications for their findings. This is particularly important for the interpretation of the Pareto analysis. The manuscript studies a potential conflict between optimizing predictions of daily incidence and optimizing predictions of categorical changes in the 7-day rolling average. As the authors of [1] point out, scoring a log transformed incidence metric rewards accurate forecasts of the epidemic growth rate (closely related to the question of predicting changes in a 7-day rolling average incidence). Given this, some of the apparent conflict between goals might be mitigated if the target for incidence scoring were log-transformed.

The choice of scoring rule is critical to evaluation of epidemic forecasting, and we agree with the reviewer the importance of selecting the right transformation. The authors of the highlighted study note in the abstract that the log transformation is one available option – and that the right transformation should be used in context. Our choice of using both an absolute and relative scoring approach reflects the varying needs of our users, which we have hopefully now more clearly outlined in the evaluation section of the methods, given below:

“Both pcWIS and RPS are transformed scoring rules with attractive properties which, when taken together, account for different forecast user preferences (cite Bosse et al). The pcWIS is an absolute metric, where periods of high values will be penalised more – which is a quality attractive to hospital managers where staff and beds have limited numbers. For example, the growth rate is less useful in this setting than knowing when the quantity of admissions will go above a threshold. The use of a per-capita transformation allows for easier comparison between locations with different population catchments in this absolute scale. The RPS penalises error of the relative prediction, which is a discretised transformation of the epidemic growth rate, which can be more informative to public health officials responsible for interventions.”

Furthermore, the authors of this work highlight that a major challenge of log transform method is in a regime where the prediction distribution has a large probability mass on zero, which is the case for our most local forecasts, and particularly during the start and end of the influenza season – though this is also a challenge when forecasting with our trend direction approach.

**2.3.5 Was individual model convergence assessed and if so how?**

Were any automated convergence checks performed on the models in real time? In the retrospective analysis? The authors note ”We see in the supplementary Figure A of retrospective forecasts that, on occasion, the GP growth rate forecasts exhibited unusual behaviour” (p. 15). I wonder whether these were symptoms of non-convergence.

All models delivered within this season were fitted using some variation of a maximise expectation algorithm rather than a Bayesian approach. As a result, we did not produce convergence checks. The specific model mentioned was experimental during the time of this paper and was not used in the later 25/26 season due to its poor performance. Note the referenced figure is now figure W.

**Minor Comments**

**3.1 Forecast pause**

The absence of operational forecasts during the period of peak national influenza incidence is an unavoidable limitation of the real-time analysis, but it should be noted and discussed, particularly given that retrospective sub-ensembles and individual models performed poorly then (Figure 6, ”Influenza forecasts were more challenging, especially when the peak was thought to be around the winter break in operations”, p. 17). Note that the poor retrospective peak performance is amplified by the decision to score per-capita incidence rather than log per capita incidence, but the RPS results suggest it is more general.

We agree with the reviewer, and reviewer 1 who also expressed similar thoughts. We have added the following to the discussion to make the point more explicitly:

“Notable however, is the Christmas winter holiday period, where there is an agreed pause with users due to changes in surveillance reporting cadences, staff availability and senior officials working. This year, this pause occurred during the peak of the influenza wave, a period of high-pressure meaning users relied on older forecasts to make decisions. While automation of modelling can improve delivery, capacity of staff trained to assure work is also a key challenge, and forecasts must align to other surveillance data cadences they rely on. The pause occurred during a time of heightened pressure, and when the influenza wave peaked – a period particularly challenging to forecast. The difficulty in this period is highlighted by the retrospective models poor performance during this time shown in Figure 6, across both pcWIS and RPS.”

**3.2 Why not compare the operational ensemble to its constituent individual operational models?**

In the manuscript, the operational (real-time) ensemble gets compared to retrospective sub-ensembles and to retrospective individual models. The authors point out that it would not be a fair comparison to compare the overall aggregate score for the operational ensemble to overall aggregate scores for individual real-time models due to the fact that some individual real-time models were only used for a subset of forecasting problems. But elsewhere (e.g. Figure 6) the manuscript shows scores for the operational ensemble by forecast date. Why not compare the operational ensemble’s scores for each date to its (real-time) constituent models from that date? Why not compare the operation ensemble overall to each of its constituent models on all and only the forecast problems where they overlap?

We agree with the reviewer and have added the operational individual models into the scoring included in Figure 2 to better contextualise the performance. We have presented all models that were available on a given forecast start date, reflecting that not all models were available at all points in time.

**3.3 Figure 2 y-axis scale**

A free y-axis for the scores plotted in Figure 2 is reasonable given that we do not necessarily expect the RPS and the pcWIS to have the same magnitudes, but I do not understand the motivation for using a log-scale y-axis for pcWIS but linear-scale y-axis for RPS. From supplement Figure D, it does not appear that there were ever predictions of the trend with complete certainty, so there should not be literal zero RPS values, and a log axis for that should be possible as well.

We thank the reviewer for highlighting this inconsistency – we have chosen to log all scores in the manuscript to allow for more intuitive reading of the figures between pcWIS and RPS.

**3.4 Figure 6: temporal resolution of scores**

In Figure 6, score timeseries for individual models and sub-ensembles appear to have more frequent changepoints than the operational ensemble score (see e.g. the worst scoring individual model (highest yellow line) by log(pcWIS) for COVID-19 in mid-January, where we appear to have more-than-weekly scores (despite the scores being displayed “prediction start date”, i.e. forecast date). Should this occur? If so, why?

After further investigation we determined that this was a result of a data processing error, which we have rectified. The figure now shows forecasts at regular intervals as expected.

**3.5 Legend readability**

Legend and axis text are very small for all figures. So are legend key glyphs. I strongly suggest making them larger to improve figure readability.

Thank you for addressing. We have improved the sizing of text in plots where possible.

**3.6 Wording suggestions**

This suggestion makes the point we were trying to make much clearer and we appreciate the suggestion; we have implemented this exactly.

p. 13: “We see gradual upward trends in the performance of trend direction estimation for ETS, cubic regression and Gaussian process models.” This is ambiguous. It could be read to imply model improvement (“upward trend in performance”). In fact, it appears to refer to the y-values in the heading upward over time. Given the meaningful of the y-axis, that in turn means worsening contributions to the ensemble (in some cases moving from helping to hurting).

We thank the reviewer for spotting this error and have updated the ambiguous (and incorrect) statement to the following:

“For RPS contribution we see worsening in the performance estimates for ETS, cubic regression and Gaussian process models over the study period”

p. 16: “That being said, our modal forecast was stable, so stakeholders received a correct forecast on the majority of weeks”. It is not clear to me what this means. Is the point that just that for the majority of forecast weeks, the highest predictive-probability category and the eventual observed value were both “stable”? Or is it a claim that there were weeks when RPS was not particular good (e.g. due to an uncertain forecast) but when the highest-predictive-probability category nonetheless ended up being to the observed category? Or something else?

We can see this is a confusing way to present results. We have now added Table 2 which shows how often the highest predicted probability category match the observed trend direction. We have also added the following text to the manuscript:

“if we treat the operational ensemble’s mostly likely trend direction at the national level matched the observed outcome on the majority of occasions: 56% of COVID-19 trend direction forecasts and 63% of Influenza trend direction forecasts matched the observed outcome. Note that the number of correct mostly likely trend direction predictions is not a proper scoring rule, but is an easy to understandable performance metric for a variety of non-technical stakeholders. Supplementary Table 2 presents results at all spatial resolutions.”
