# Supplementary material for: Evaluation of short-term multi-target respiratory forecasts over winter 2024-25 in England using sub-ensemble contribution analyses
Source: PLoS Comput Biol. 2026 Aug 12;22(8):e1014644. doi: 10.1371/journal.pcbi.1014644 (PMC13506087; doi:10.1371/journal.pcbi.1014644)
Supplement: Supplementary file 36 [file pcbi.1014644.s037.docx]

**Response to Round 2 review**

We thank both reviewers for their engagement with this work and helpful feedback. We have addressed all remaining comments in full.

Reviewer #1

The major concerns I had were mostly clarifying methods and expanding on or including discussion points that I thought were missing. The authors have done a good job responding to these comments. After reading through the responses to my comments (and the other reviewer comments) it is clear the authors made a good faith effort to revise the manuscript.

We thank the reviewer for the positive feedback on the revised manuscript and the time taken to engage with the work. We appreciate their constructive feedback and feel the quality of the research has improved thanks to their suggestions.

The only additional request to complete Supplementary Table 2. It is supposed to have results but it is only a list of models used in the full Pareto analysis.

Regarding supplementary table 2, we agree this could have been presented more clearly. We have reworded the caption and one column heading to clarify the content of this table; the column refers to the sub-ensembles which are on the Pareto Front (rather than the set of all models used in the analysis). The front is quite large in this case. This table therefore contains the results of the pareto analysis.

Reviewer #2:

The updated manuscript addresses my concerns and those of the other reviewer. Key changes include:
 - A more comprehensive results section (e.g. predicted/actual plots and forecast scores at sub-national levels)
 - More detail about forecast models and evaluation methods
 - An added analysis comparing the operational ensemble to individual operational models and to a matched retrospective ensemble

I believe the manuscript is now publishable. I have a few remaining minor scientific concerns, but I believe these can be addressed without re-review. I have also flagged some typos.

Thank you for the comments and feedback on the revised manuscript. We appreciate the additional feedback to bring this to publication quality.

All page numbers refer to the compiled review PDF (including frontmatter).

\## Remaining minor scientific concerns
 Supp Table 1: Supplementary Table 1 is cited to justify the claim that "the ensemble was on average, provided both better numerical forecasts and trend direction forecasts of COVID-19 and Influenza at all spatial resolutions" (p. 39). To fully justify this claim (and fully address my prior comments), the table should include the seasonal average scores for each individual (retrospective) model (i.e. seasonal average scores for each of `ets,` `gam_cr`, etc.) in addition to the average score across individual (retrospective) models.

The table has been reformatted and now contains mean season scores for all the individual models (as well as all the data in the previous version of the paper). We have also changed the text in the paper to

*“this shows that ensembles (either operational or matched) usually provide the best numerical or trend direction forecast of COVID-19 and Influenza. In 8 out of 12 combinations (2 diseases, 2 metrics, 3 location levels) an ensemble is the best model. Ensembles were better than the majority of individual models in the cases where the ensemble was not optimal, see Supplementary Table 1 for all mean score values by model across the season.”*

p. 43: "The pcWIS values for the operational ensemble are usually around the average for retrospective sub-ensembles; over the season the average pcWIS values for COVID-19 and Influenza operational ensemble forecasts were 4.69 × 10−7 and 5.62 × 10−7 respectively." The logic in this sentence is a bit unclear. The first clause makes a comparison between the operational ensemble and the average of retrospective sub-ensembles with some kind of time stratification ("usually"). The second clause reports an overall value for the whole season, only for the operational ensemble. These are separate points, and should probably be separate sentences. If the first point ("The pcWIS values for the operational ensemble are usually around the average for retrospective sub-ensembles") is to be retained, the authors must reference a figure or table that demonstrates it.

We have updated the text to

*“The pcWIS values for the operational ensemble are on the same scale as the pcWIS values for the retrospective sub-ensembles (Figure 6). Over the season the average pcWIS values for COVID-19 and Influenza operational ensemble forecasts were 4.69 × 10−7 and 5.62 × 10−7 respectively”*

Supp Fig Q: Since 50% coverage was also assessed in the manuscript alongside 90% coverage, should include a version of this plot for 50% coverage as well.

We are happy to implement this suggestion especially as the 50% interval is shared with forecast users. Figure Q has been updated to show both the 90% and 50% coverage statistics.

Supp Fig Q: Is the line the overall average coverage over locations _and_ target dates? It should be. Should revise to make it so if not, and should define the line in the caption.

We appreciate the original figure was unclear about this. The revised figure (with 50% coverage) now explains in the subtitle that the blue line is the nominal coverage and orange lines are mean coverages across all locations for a given location level on a specific prediction start date (for example, the mean coverage across ICB locations).

Supp Fig X: Is the outlier ICB with an unusually bad pcPCS but fairly typical RPS for COVID-19 possibly due to a scoring bug? Should investigate. If not due to a bug, the outlier should be noted and its cause explained to the extent possible. Optional related suggestion: perhaps worth making a version of the plot with that point excluded so that the trend for COVID excluding it is clearer. That would also enable plotting flu and COVID with shared x and y axis scales, which would also help the reader determine whether the strength of the association is stronger for one versus the other.

We investigated the data and found that for this ICB, the last week of data was entirely zeros. This resulted in a very poor score for the final forecast, as the prediction intervals did not extend to zero. The other scores for this ICB across the season were of a similar order of magnitude to other ICB pcWIS values (between -16 and -12 once logged). The outlier forecast had a log pcWIS of about -1. We have extended the captions to provide context for the outlier. We have also taken on board the optional suggestion and expanded the ytfure to include a version of the scatterplot with the outlier removed, and all data on axes with the same scale.

We have therefore revised the figure caption:
“Bubble chart showing log(RPS) vs log(pcWIS) for different forecasting locations, point size represents the population of the location for forecasting. (Middle) Same bubble chart with an outlier removed; there is an outlier ICB for COVID-19 with a log(pcWIS) value of approximately -3.5. Upon further investigation, we saw the final week of data for this ICB reported zero admissions every day. It is unclear if this is a poor forecast or a data quality issue, as admissions counts in the very late season are often very low (below 5 admissions per day).) Boxplot showing population sizes per location level. In general, scores are lower (better) for locations with a larger population. (Bottom) Boxplot showing population sizes per location level. “

Supp Table 4: There is a problem with this table (possibly due to a code bug?). The mean retrospective ensemble scores should lie between the min and max scores, but do not always (e.g. COVID-19 pcWIS mean is greater than max, COVID-19 RPS mean is less than min).

Thank you for bringing this to our attention. This has been investigated (we believe it was a manual data transfer copying error) and the values have been revised. We now have the overall mean sitting between the minimum and maximum for all columns.

\## Typos and minor wording points

Thank you for identifying these minor wording points, we have addressed them all to improve the readability of the work. We have addressed each one in turn.

p. 25: "We compared operational retrospective forecasts". Missing word(s). Should be "We compared operational forecasts to retrospective forecasts" or "We compared operational and retrospective forecasts".

We have removed the word “operational” from this sentence, as it was just the retrospective forecasts being analysed here.

p. 26: "The sub-ensemble simulation showed individual models influenced the ensembles during different epidemic phases." I am not 100% sure what the intended point is here. Individual models always have some impact on an ensemble's forecast and its performance. Is the point here is that individual models often had a strong effect (good or bad) on ensemble performance in one epidemic phase but a weak effect in another? If so, that should be made more explicit.

Reworded to “The sub-ensemble simulation showed how individual models influenced the ensemble scores during different epidemic phases”

p. 26: "single, more accurate" should be "single, ideally more accurate" or something similar. Need to acknowledge that ensembling does not necessarily succeed in its goal of improving predictions.

We have now included the word “ideally”.

Fig 1: typo in legend: "operation" should be "operational".

We have corrected the typo in the plot.

p. 39: Extra word(s) in this clause: "this shows that the ensemble was on average, provided both better numerical forecasts and trend direction forecasts of COVID-19 and Influenza at all spatial resolutions." I think this should be: "the ensemble on average provided both better numerical forecasts and better trend direction forecasts of COVID-19 and Influenza at all spatial resolutions".

We have used the suggested wording in the revised manuscript.

p. 43 extra words: "That being said, if we treat the operational ensemble's mostly likely trend direction at the national level matched the observed outcome on the majority of occasions". I think this should be simply: "That being said, the operational ensemble's mostly likely trend direction at the national level matched the observed trend direction on the majority of occasions".

We have updated the wording of the manuscript to the suggested wording.

p. 46: "As with any scoring-based approaches". "Approaches" should be "approach".

We have changed “approached” to “approach”

Supp Fig F, G caption: Use "improved performance" instead of "increasing performance" in these captions (avoids confusion due to negatively-oriented scores, etc.).

We have now used the work “improved” instead of “increased”

Supp Fig G: Figure title is incorrect. Says "pcWIS for regional RSV forecasts" but is actually pcWIS by age group for national forecasts.

The title now refers to “age breakdowns” rather than “regional” dissaggregations

Supp Fig Q: Typo in figure title. Title should be "Observed versus theoretical coverage" or "Observed versus nominal coverage".

Title is now “Observed vs Nominal Coverage Statistics”

Supp Fig U: Typo in spelling of "estimate" in the caption.

We have corrected the spelling of “estimate”.
